# Mental, physical, and respiratory health in people with tuberculosis in Southern Africa: a multi-country cohort analysis

**DOI:** 10.1186/s12916-025-04321-6

**Published:** 2025-08-20

**Authors:** Nicolas Banholzer, Guy Muula, Fiona Mureithi, Denise Evans, Jacqueline Huwa, Idiovino Rafael, Cordelia Kunzekwenyika, Nelly Jinga, Amina Fernando, Agness Thawani, Remo Schmutz, Carolyn Bolton, Gunar Günther, Matthias Egger, Andreas D. Haas, Annika C. Sweetland, Marie Ballif, Lukas Fenner, IeDEA Southern Africa (IeDEA-SA)

**Affiliations:** 1 Institute of Social and Preventive Medicine (ISPM), University of Bern, Bern, Switzerland; 2 Centre for Infectious Disease Research in Zambia (CIDRZ), Lusaka, Zambia; 3 Health Economics and Epidemiology Research Office, Faculty of Health Sciences, University of the Witwatersrand, Johannesburg, South Africa; 4 Lighthouse, Lilongwe, Malawi; 5 SolidarMed, Chiure, Mozambique; 6 SolidarMed, Masvingo, Zimbabwe; 7 Department of Pulmonology, Allergology and Clinical Immunology, Inselspital, University Hospital of Bern, Bern, Switzerland; 8 Centre for Integrated Data and Epidemiological Research (CIDER), School of Public Health, University of Cape Town, Cape Town, South Africa; 9 Bristol Medical School, Population Health Sciences, University of Bristol, Bristol, UK; 10 Department of Psychiatry, Columbia Vagelos College of Physicians and Surgeons/New York State Psychiatric Institute, New York, NY USA; 11 Department of Infectious Diseases, Inselspital, Bern University Hospital, University of Bern, Bern, Switzerland; 12 Department of Infectious Diseases and Hospital Epidemiology, University Hospital Zurich, University of Zurich, Zurich, Switzerland

**Keywords:** TB, Quality of life, Mental health, Physical fitness, Depression, Post-TB complications, Low- and middle-income countries, Africa, HIV, Gender

## Abstract

**Background:**

Tuberculosis (TB) affects people’s quality of life (QoL). We prospectively monitored physical and mental health-related QoL over time in people with TB in the Southern African region with a high HIV and TB burden.

**Methods:**

Adults aged ≥ 15 years with pulmonary TB were enrolled in five cohorts in Malawi, Mozambique, South Africa, Zambia, and Zimbabwe from October 2022 to September 2024. We assessed six QoL outcomes using validated instruments at the start (baseline), end of treatment, and 6 months post-treatment: symptoms of depression (PHQ-9), mental and physical health (SF-12 mental, SF12-MC, SF-12 physical component, SF12-PC), physical fitness (6-Minute Walk Test, 6MWT; 1-min Sit-To-Stand Test, STST), and respiratory health (Saint-George-Respiratory-Questionnaire, SGRQ). Missing QoL scores were imputed with multivariate imputation by chained equations. We compared the proportion of participants with impaired QoL, defining impairment based on outcome-specific cut-off values. We also estimated changes in QoL scores and examined their associations with baseline characteristics using Bayesian multivariable regression models.

**Results:**

We included 1438 participants with a median follow-up of 344 days (interquartile range [IQR] 183–373). The median age was 39 years (IQR 30–50); 67% were male, and 39% living with HIV. At baseline, 49% had symptoms of depression, 73% had impaired mental health and 92% impaired physical health-related QoL, 68–74% had reduced physical fitness (68%: 6MWT, 74%: STST), and 78% impaired respiratory health. All QoL outcomes improved by the end of treatment, notably depressive symptoms (48% to 5%), mental health-related QoL (73% to 28%), and respiratory health (78% to 11%). Most QoL impairments continued to decrease post-treatment, especially physical and respiratory health; depressive symptoms remained below 5%. Across QoL domains and study visits, better outcomes were associated with age < 30 (83% probability), and worse outcomes with female gender (86%) and a prior TB history (89%). Living with HIV and alcohol drinking were associated with worse QoL only at baseline (88% and 87%).

**Conclusions:**

TB negatively impacts QoL across physical, mental, and social domains, including post-treatment. The study highlights the need for integrated mental and physical healthcare and rehabilitation during TB treatment and beyond, especially for high-risk populations, to address the long-term impact of TB on QoL.

**Supplementary Information:**

The online version contains supplementary material available at 10.1186/s12916-025-04321-6.

## Background

After the COVID-19 pandemic, tuberculosis (TB) has again become the leading cause of death from infectious diseases worldwide, surpassing HIV [1]. In 2023, an estimated 10.8 million people fell ill with TB globally, resulting in 1.25 million deaths, including 161,000 people living with HIV [1]. The chronic disease, stigma, discrimination, and economic impact associated with TB contribute to a deterioration in the quality of life (QoL) of people with TB, both physically and mentally [2–5]. In addition, people who have completed TB treatment may continue to suffer from long-term physical and mental complications—an additional burden that has long been underestimated and remains poorly described [2, 6, 7].

Studies have shown that the QoL of people with TB is generally worse than that of the general population [4, 8]. Indeed, mental health disorders such as depression and anxiety are highly prevalent among people with TB [9, 10], especially among those with multidrug-resistant (MDR) TB [11]. Furthermore, post-TB lung disease defined as a chronic respiratory abnormality at least partially attributable to previous pulmonary TB [2, 12] is estimated to affect 60% to 90% of people who were treated for TB, affecting their QoL, including physically and mentally [12]. In terms of mortality, people treated for TB have an almost threefold increased risk of dying after treatment compared with matched controls [13]. Altogether, the post-TB complications in terms of morbidity, mortality, and impaired QoL contribute significantly to the overall burden of TB disease. There is a need for a better understanding of these complications that integrates both mental and physical health aspects [14, 15]. Existing studies often lack comprehensive longitudinal data that track multiple aspects of QoL from the initiation of treatment to the post-treatment period across diverse settings. Here, we investigated the evolution of physical and mental health-related QoL before, during, and after TB treatment in a prospective cohort of people with TB in five high-burden HIV and TB countries in Southern Africa.

## Methods

### Study design

In a prospective, non-interventional cohort study, we consecutively enrolled people with pulmonary TB from five HIV care clinics and their associated TB clinics in the International epidemiology Databases to Evaluate AIDS in Southern Africa (IeDEA-SA).

### Study setting

We prospectively enrolled people with TB at the following study sites: Centre for Infectious Disease Research in Zambia, Lusaka, Zambia; Themba Lethu Clinic, Helen Joseph Hospital, Johannesburg, South Africa; Lighthouse and Martin Preuss Center, Lilongwe, Malawi; Chiure Health Center, Mozambique; Maswingo Health Center, Zimbabwe.

### Study population

Between October 2022 and March 2023, we recruited people diagnosed with pulmonary TB aged ≥ 15, living with or without HIV, and with or without additional extrapulmonary manifestations (e.g. lymph nodes, pleura). We included the cohort data available on January 30, 2025. TB was either bacteriologically confirmed (e.g. using Xpert Ultra or lipoarabinomannan urine tests) or clinically diagnosed based on clinical symptoms and chest X-ray results. TB treatment and other diseases, including mental health, were managed according to local guidelines. We followed cohort participants with study visits at the time of TB treatment start (baseline), end of treatment (usually 6 months after treatment start), and 6 months after treatment completion (post-treatment). We made every effort to ensure the retention of the study participants. This included regular phone calls from the study team, reimbursement for travel and time in accordance with local ethics committee recommendations, tracing teams, community support, and support from local clinic staff at the TB clinics.

### Data collection

Trained health workers collected patient data at each study visit using standardised questionnaires [16]. We measured six health-related QoL outcomes at each study visit using a comprehensive set of instruments that are validated for TB research and are available in local languages: depressive symptoms were assessed using the Patient Health Questionnaire-9 (PHQ-9) [17], mental and physical health using the two subscales of the 12-Item Short Form Health Survey (SF-12; Mental Component Summary, SF12-MCS; Physical Component Summary, SF12-PCS) [18], physical fitness using objective scores of the 6-Minute Walk Test (6MWT) [19] and the 1-min Sit-to-Stand Test (STST) [20], and respiratory health using the St. George Respiratory Questionnaire (SGRQ) [21]. An overview of the QoL instruments and outcomes used in our study is provided in Table 1. All data were collected electronically on the secure web platform REDCap [22].

**Table 1:** Summary of standardised questionnaires used in the study to measure quality of life (QoL)

Outcome	Instrument name (reference)	Description	Score (interpretation)	Cut-off
Symptoms of depression	PHQ-9: Patient Health Questionnnaire-9 [17]	Self-reported tool to assess common mental health disorders, such as depression and anxiety	0–27 (lower scores are better)	≥ 7
Impaired mental health	SF12-MCS: SF-12, Mental Component Summary [18]	The SF12-MCS is a subscale of the SF12^a^ that measures mental health-related quality of life, focussing on emotional well-being, social functioning, vitality, and the psychological impact of health conditions	0–100 (higher scores are better)	< 50
Impaired physical health	SF12-PCS: SF-12, Physical Component Summary [18]	The SF12-PCS is a subscale of the SF-12^a^ that evaluates physical health-related quality of life, focussing on physical functioning, body pain, general health, and the impact of physical health on daily activities	0–100 (higher scores are better)	< 50
Physical fitness	6MWT: 6-Minute Walk Test [19]	The 6MWT is a simple and standardised method to evaluate the cardio-pulmonary functional exercise capacity in patients with pulmonary diseases. The 6MWT is performed along a flat, straight, enclosed corridor in or near the clinic	Distance in m (longer distances are better)	< 400 m
Physical fitness	STST: Sit-to-Stand Test [20]	Functional assessment tool that evaluates lower limb strength, endurance, and functional capacity by counting the number of times an individual can rise from a seated position to standing and return within 1 min	Number of cycles per minute (more cycles are better)	< 20 cycles
Impaired respiratory health	SGRQ: St. George Respiratory Questionnaire [21, 38]	Self-administered tool to assess health-related quality of life in individuals with respiratory diseases, measuring the impact of symptoms, activity limitations, and the overall burden of the disease	0–100 (lower scores are better)	< 25

^a^The 12-Item Short Form Health Survey (SF-12) is a self-reported and shortened version of its predecessor, the SF-36, which evolved from the Medical Outcomes Study. The SF-12 was created to reduce the burden of response. It uses the exact eight domains as the SF-36. There is a Mental Component Score (SF12-MCS) and a Physical Component Score (SF12-PCS)

### Definitions

We defined depressive symptoms using a cut-off of PHQ-9 ≥ 7 according to validation studies conducted in three of the five participating countries (South Africa, Malawi, Mozambique) [23–26]. However, we conducted a sensitivity analysis using the standard PHQ-9 cut-off of 10 for depressive symptoms, which also corresponds to the cut-off used in Zimbabwe [17, 27]. All other cut-offs used are shown in Table 1. Alcohol drinking and tobacco smoking were defined as any current use with an Alcohol, Smoking and Substance Involvement Screening Test (ASSIST) score greater than zero in the appropriate score category. Serious non-fatal adverse events were considered if the event led to a persistent disability, medical consultation, or an in-patient hospitalisation. Multidrug-resistant TB (MDR TB) was defined as resistance to at least rifampicin.

### Statistical analyses

We analysed demographic and clinical characteristics at baseline and compared QoL outcomes between study visits. Some QoL scores were missing if an outcome was not recorded during a visit, the patient died before a follow-up visit, or the patient did not return for a follow-up visit within 8 months (16 months for people with MDR) of the last visit. Planned visits of participants who did not yet reach the time of the scheduled follow-up were excluded. Missing scores of other visits were imputed using multiple imputation by chained equations based on QoL scores and baseline patient characteristics. We generated 20 datasets during imputation. In addition, we calculated standardised QoL scores (*z*-scores) in each dataset to make our estimation results comparable for continuous scores measured on different scales. Before standardisation, PHQ-9 and SGRQ scores were transformed by subtracting the measured value from the maximum possible value, so that positive changes represent an improvement in QoL for all outcomes.

We first compared the proportion of study participants with impaired QoL (as defined above) by study visit separately for each outcome using Bayesian binomial models. Then, we compared the continuous QoL *z*-scores by study visit and their association with patient characteristics using Bayesian linear mixed-effects models with time interaction effects, adjusting for mental health treatment since the last study visit and any non-fatal serious adverse event during treatment. The time effects were used to compute the estimated changes in QoL *z*-scores. The associations of higher (or lower) QoL *z*-scores by study visit with patient characteristics at baseline were reported as probabilities by calculating the proportion of posterior draws with a positive (or negative) estimate. To investigate whether some patient characteristics were more strongly associated with low QoL scores at the end of treatment, we estimated their association with baseline patient characteristics using Bayesian quantile regression models, adjusting for baseline QoL scores.

We present the results using the observed data and the imputed data for unrecorded outcomes, excluding missing follow-up visits and deaths. In Additional file 1, we present the results of the complete case analyses, i.e. considering only recorded outcomes. In sensitivity analyses, we show the changes in QoL when including the fully imputed scores for study visits where the patient died before or did not return for a follow-up visit (all QoL scores missing). Estimation results (posterior samples of each model parameter) were pooled and summarised with medians and 95%-credible intervals (CrIs) or the posterior probability (Pr in %). All analyses were performed in R version 4.3.2 and probabilistic modelling in Stan version 2.26.1 using the brms R package version 2.20.4.

## Results

### Patient characteristics and QoL at the start of TB treatment

We included 1438 study participants with TB (Table 2) who were enrolled and followed between 2022 and 2025, for a total follow-up of 405,923 person-days with a median follow-up of 344 days per patient (interquartile range [IQR] 183–373). All participants started TB treatment (baseline); 1164 (81%) came for the end of treatment, and 745 (52%) for a follow-up visit 6 months post-treatment (see Additional file 1: Table S1). Overall, 20% of study participants were clinically diagnosed with TB.

**Table 2:** Patient characteristics of study participants at the time of tuberculosis (TB) treatment start, overall and by country of the study site

Variable *n* (% of non-missing observations)	All *n* = 1438	South Africa *n* = 222	Malawi *n* = 300	Mozambique *n* = 300	Zambia *n* = 361	Zimbabwe *n* = 255
Median age (IQR), years	39 (30–50)	40 (33–49)	35 (28–45)	47 (34–58)	34 (28–42)	44 (34–57)
Male gender	963 (67)	139 (63)	226 (75)	168 (56)	275 (76)	155 (61)
History of prior TB	251 (17)	42 (19)	68 (23)	3 (1)	97 (27)	41 (16)
Cavitary disease	390 (27)	24 (12)	111 (37)	43 (51)	115 (34)	97 (49)
MDR TB^a^	59 (4)	11 (5)	1 (< 1)	0 (0)	40 (11)	7 (3)
Type of diagnosis						
Bacteriologically confirmed	914 (80)	207 (93)	171 (57)	NA	346 (96)	190 (75)
Clinically diagnosed	224 (20)	15 (7)	129 (43)	NA	15 (4)	65 (25)
TB manifestation						
Pulmonary only	1411 (98)	217 (98)	279 (93)	300 (100)	360 (> 99)	255 (100)
Pulmonary and extrapulmonary	22 (2)	0 (0)	21 (7)	0 (0)	1 (< 1)	0 (0)
HIV infection						
Negative	859 (60)	83 (37)	191 (64)	218 (73)	227 (63)	140 (55)
Positive	563 (39)	129 (58)	108 (36)	78 (26)	134 (37)	114 (45)
CD4 cell count/mm^3^, median (IQR)	256 (139–433)	202 (69–321)	224 (144–327)	NA	318 (164–481)	488 (276–700)
HIV unknown	16 (1)	10 (5)	1 (< 1)	4 (1)	0 (0)	1 (< 1)
BMI^b^						
Underweight	521 (38)	54 (27)	140 (47)	123 (42)	148 (41)	56 (28)
Normal	806 (60)	135 (67)	152 (52)	169 (58)	211 (58)	139 (69)
Obese	25 (2)	12 (6)	3 (1)	1 (< 1)	2 (1)	7 (3)
Any current tobacco smoking	497 (57)	75 (35)	108 (72)	79 (87)	176 (59)	59 (53)
Any current alcohol drinking	626 (67)	100 (47)	150 (77)	48 (76)	242 (76)	86 (63)
Any mental health treatment^c^	13 (2)	3 (5)	4 (2)	0 (0)	3 (1)	3 (1)

*BMI*, body mass index; *IQR*, interquartile range; *MDR*, multidrug-resistant; *NA*, not available; *TB*, tuberculosis

^a^Resistance to at least rifampicin

^b^Obese (BMI > 30 kg/m^2^), normal (BMI ≥ 18 kg/m^2^ and ≤ 30 kg/m^2^), underweight (BMI < 18 kg/m^2^)

^c^Any mental health treatment (counselling, hospital admission, or any medication for a mental illness during TB treatment and beyond)

**Fig. 1:**
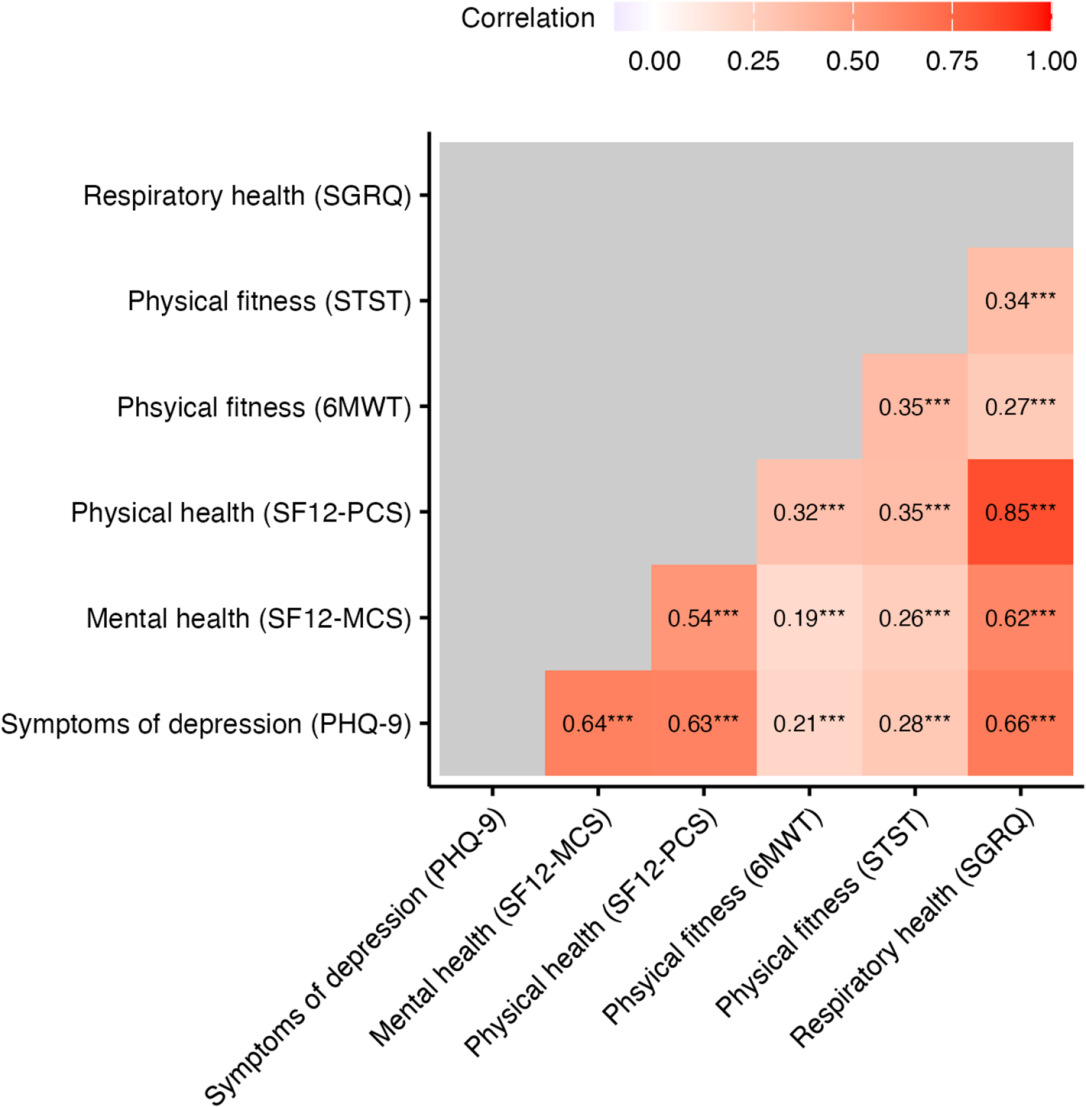
Pairwise correlations between quality of life (QoL) health scores across time points. Stars indicate significance levels: *p* < 0.05 (*), *p* < 0.01 (**), *p* < 0.001 (***). See Supplementary Fig. 1 for correlations stratified by study visit. 6MWT, 6-Minute Walk Test; PHQ-9, Patient Health Questionnnaire-9; SF-12, Short Form Health Survey: Mental Component Score (SF12-MCS), Physical Component Score (SF12-PCS); SGRQ, St. George Respiratory Questionnaire; STST, Sit-to-Stand Test

At baseline, the median age was 39 years (IQR 30–50), 963 (67%) were men, 563 (39%) were living with HIV, 251 (17%) reported a history of TB; 59 (4%) had MDR TB; 497 (57%) reported smoking tobacco, and 626 (67%) drinking alcohol (Table 2). Only 13 (2%) participants reported receiving any mental health treatment between study visits. Depressive symptoms were present in 677 (47%) participants, 1026 (71%) had impaired mental health-related QoL, 1288 (90%) impaired physical health-related QoL, 590 (41%, 6MWT) and 728 (51%, STST) had lower physical fitness than expected for a healthy adult, and 1113 (77%) had impaired respiratory health. QoL scores were often correlated, e.g. SF12-PCS with SGRQ (Pearson’s *r* = 0.85, *p* < 0.001), or PHQ-9 with SF12-MCS (*r* = 0.64, *p* < 0.001; Fig. 1, Additional file 1: Fig. S1).

### Changes in QoL over time

**Fig. 2:**
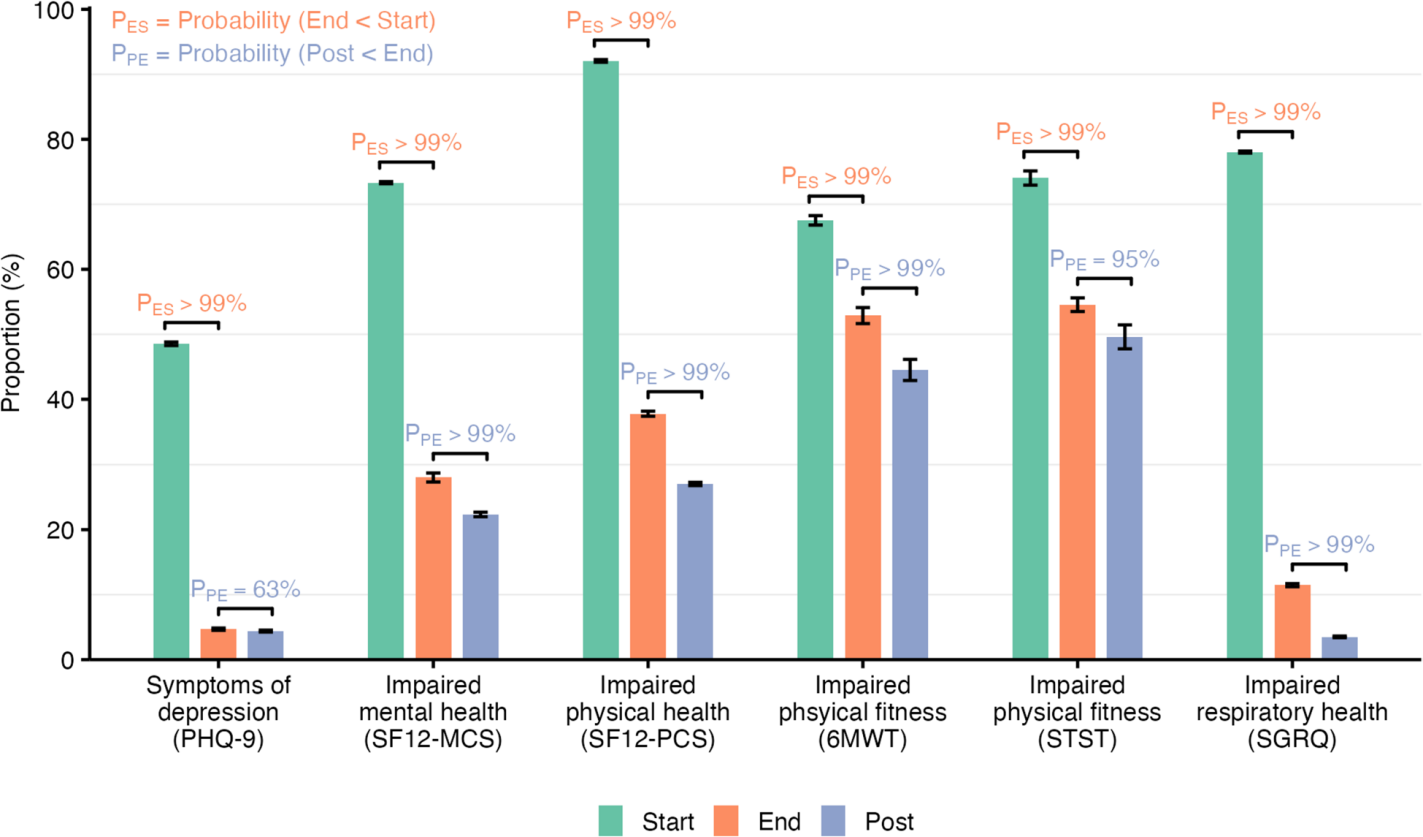
Estimated proportion (in %) of patients with impaired QoL. Bars show the mean proportion and error bars the standard deviation. Bars are annotated with the posterior probability that the proportion of patients with impaired QoL is lower at the end versus the start of TB treatment (P_ES_), and the probability that it is lower 6 months post-treatment versus the end of treatment (P_PE_). 6MWT, 6-Minute Walk Test; PHQ-9, Patient Health Questionnnaire-9; SF-12, Short Form Health Survey: Mental Component Score (SF12-MCS), Physical Component Score (SF12-PCS); SGRQ, St. George Respiratory Questionnaire; STST, Sit-to-Stand Test

The prevalence of participants with impaired QoL decreased by the end of treatment (Fig. 2). The largest decreases were observed for depressive symptoms and mental health-related QoL (PHQ-9: 49% to 5%; SF12-MCS: 73% to 28%), physical health-related QoL (SF12-PCS: 92% to 38%), and respiratory health (SGRQ: 78% to 11%). The prevalence of depressive symptoms remained low post-treatment (4%). Mental health (SF12-MCS: 28% to 22%), physical health (SF12-PCS: 38% to 27%), and respiratory health (SGRQ: 11% to 4%) impairments continued to decrease post-treatment compared to the end of treatment. The prevalence of impaired physical fitness decreased less (6MWT: 68% to 53% to 45%, STST: 74% to 55% to 50%). A sensitivity analysis using the higher cut-off for depressive symptoms of PHQ-9 ≥ 10 (instead of ≥ 7) showed similar changes over time (Fig. S2). Changes in QoL over time were consistent across countries (Fig. S3). The prevalence of impaired QoL and temporal trends were also similar in the complete case analysis (Fig. S4). The trajectories of participants with depressive symptoms are shown in Fig. S5. A similar proportion of people with depressive symptoms at baseline returned for their end-of-treatment visits (604 out of 677, 89%) compared to individuals without depressive symptoms (649 out of 716, 91%).

The continuous QoL scores by study visit are shown in Fig. S6 (values in Table S2). The estimated changes in QoL scores mirrored the changes in the prevalence of QoL impairment, with an average increase of more than one standard deviation for mental, physical, and respiratory health scores and a smaller increase of less than half a standard deviation for physical fitness scores (Fig. S7). The estimated changes were similar when missing follow-up visits and visits of deceased people were included (Fig. S8). Further, the increases in QoL observed in study participants with follow-up visits were unlikely to be offset by potential decreases in participants with missed follow-up visits (Fig. S9).

### Patient characteristics associated with QoL scores over time

**Fig. 3:**
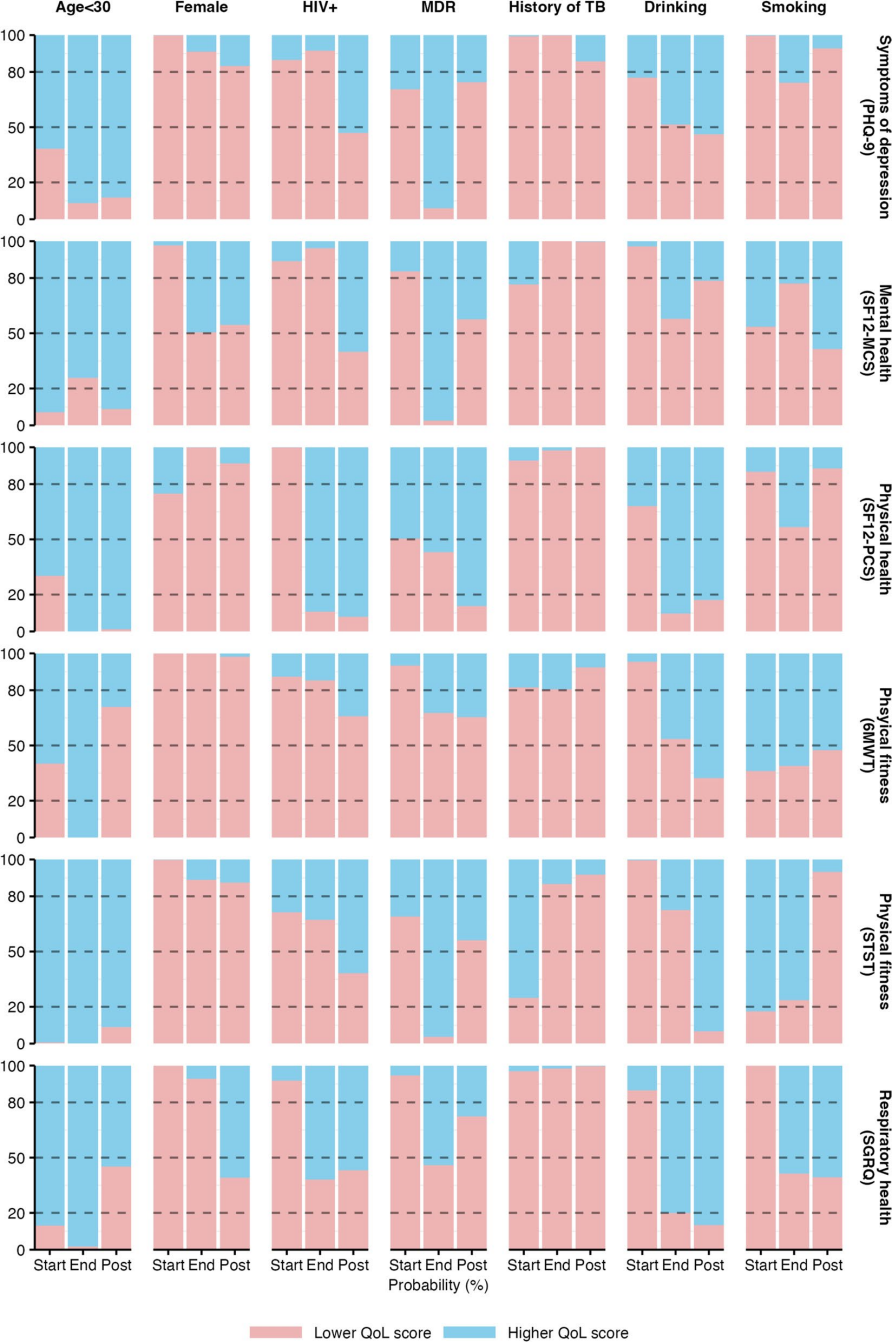
Probability that patient characteristics are associated with lower (red) or higher (blue) QoL health scores. Probabilities are computed as the proportion of posterior draws with a negative (red) or positive (blue) estimate. PHQ-9 and SGRQ scores were transformed so that for all outcomes, a positive estimate indicates a positive association of the patient characteristic with QoL. See Supplementary Table 3 for numerical results. 6MWT, 6-Minute Walk Test; PHQ-9, Patient Health Questionnnaire-9; SF-12, Short Form Health Survey: Mental Component Score (SF12-MCS), Physical Component Score (SF12-PCS); SGRQ, St. George Respiratory Questionnaire; STST, Sit-to-Stand Test

Figure 3 shows the adjusted Bayesian probability that patient characteristics were associated with positive or negative changes in QoL *z*-scores (values in Table S3). Age < 30 was associated with higher QoL scores across all domains at baseline, end of treatment, and post-treatment (overall probability across study visits and QoL outcomes: 83%). Female gender (overall probability of 86%) and a history of TB (89%) were associated with lower levels of all QoL outcomes across study visits. At baseline, living with HIV and MDR TB were associated with lower QoL scores (overall probabilities for all QoL outcomes: 88% and 77%), but at the end of and post-treatment, these associations were mixed, with some trend reversals towards improvements over time. Drinking alcohol and smoking tobacco were associated with more depressive symptoms at baseline (PHQ-9: 77% and > 99%).

### Subsequent adverse events, death, and loss to follow-up and associations with QoL scores

Across domains, QoL *z*-scores were lower at baseline for study participants who died during treatment (Table S4) and mostly for participants who had a non-fatal serious adverse event (except PHQ-9 and STST, Table S5). There was a small tendency that baseline QoL scores were lower for participants without an end-of-treatment visit (Table S5).

### Persistently low QoL and associations with patient characteristics

We explored the characteristics of individuals whose QoL did not improve at the end of treatment using quantile regression (lowest quantiles Q10 and Q25 in red represent participants with persistently low QoL, Fig. S10). Across all QoL domains (mental, physical, and respiratory health), persistently low QoL scores were more frequent in women and participants with a history of TB. Persistently low scores for depressive symptoms and mental health (PHQ-9, SF12-MCS) were more frequent in people with HIV and less frequent in people with MDR TB. Persistently low scores for physical health and fitness scores (SF12-PCS, 6MWT, STST) were more frequent in people aged 30 years or older.

## Discussion

In this longitudinal multi-country cohort study, more than 1400 people treated for TB, 40% with HIV, in five countries in the Southern African region were prospectively followed from treatment initiation to post-treatment periods using a wide range of longitudinally repeated mental and physical health-related QoL measures. We observed that the mental, physical, and respiratory health-related QoL were substantially impaired at the time of treatment initiation. All QoL outcomes improved during TB treatment and most continued to improve post-treatment. We identified vulnerable groups at higher risk of impaired QoL, especially women and people with a history of TB.

We found that multiple dimensions of mental and physical QoL outcomes were impaired in people with TB at treatment initiation and improved during treatment. Symptoms of depression and mental health scores remained low and other measures continued to improve post-treatment. The results were consistent when analysing fixed cut-offs and continuous data and were comparable across countries. The persistence of physical impairment at the end of treatment may reflect the fact that lung function impairments take longer to resolve, often up to 12–18 months after diagnosis [28], even though respiratory symptoms have been shown to resolve more quickly [29]. Our findings are consistent with a study in South Africa where QoL was reported as poor at treatment initiations, with improvements during treatment [30]. A study conducted in Malaysia showed that physical and mental QoL scores of people with TB remained worse than within the general population throughout treatment, with a persisting higher risk of depression at the end of treatment [4]. A cross-sectional study in South Africa reported that the mean summary scores obtained for mental and physical health were lower than the population norm in post-TB patients [8].

Almost half of our study participants had depressive symptoms at treatment initiation when using a region-specific PHQ-9 cut-off ≥ 7 [23–26], and less than 25% when using the universal PHQ-9 cut-off ≥ 10 [17, 27]. A meta-analysis estimated that 45% of people with TB experience symptoms of depression at the start of treatment [9]. We also showed that almost 75% of our participants had an impaired mental health-related QoL at baseline, indicating the importance of integrating mental health care for people with TB. Large reductions in impaired QoL during TB treatment were observed for mental health-related QoL and depressive symptoms, which is consistent with a smaller observational study in Peru [31]. We did not observe any further improvement in participants’ depressive symptoms, although the symptom scores were already at a low level by the end of treatment. Only a few participants reported receiving any mental health treatment during the study period. A persistently low mental health-related QoL at the end of TB treatment was more frequently reported among women and people with a history of TB.

We found that certain individual factors had an impact on QoL in people with TB, including age, gender, prior history of TB, living with HIV, and MDR TB. Overall, persistently low QoL (lowest quintiles of the scores at the end of treatment) was associated with female gender and a history of TB. The association between previous TB episodes and worse QoL scores is consistent with a study in the USA that reported a cumulative negative impact of repeated previous TB episodes on physical and mental well-being [5]. Our finding that women with TB had worse mental and physical QoL may reflect gender-related health inequities, which may be particularly severe in a context of highly stigmatised diseases such as TB and HIV, and for which underlying socioeconomic challenges prevail. The association between gender and TB-associated QoL impairment is consistent with previous research [32–35], although most were limited by their sample size or had shorter follow-up periods. Younger age has previously been associated with better outcomes on certain QoL scores (WHOQOL-BREF or SF-36) during treatment in single-country studies in Asia [32, 33, 35]. In our study, living with HIV was associated with lower mental and physical QoL at baseline, but not at the end of treatment and post-treatment. We also found that QoL was lower in people with MDR TB than in those with drug-susceptible TB. However, these differences were inconsistent over time. Some associations observed at baseline disappeared or even reversed during follow-up, potentially suggesting the balancing benefits of individual resilience and rehabilitation among people with MDR TB. In contrast, a study from urban South Africa showed that health-related QoL was lower in MDR compared with non-MDR TB [36].

Our study has limitations. First, the participating sites in IeDEA Southern Africa may not be fully representative of tuberculosis and HIV care at the national or regional level. However, the diversity between sites ensures that our data broadly reflect prevailing trends in high TB and HIV burden settings in sub-Saharan Africa. Second, we acknowledge that loss to follow-up is an inherent challenge in longitudinal studies, particularly in African settings where migration is high. Although some residual bias may remain, our sensitivity analysis indicated that only an implausibly severe worsening of QoL scores among those lost to follow-up would have offset the observed improvements. Third, QoL instruments such as PHQ-9 and SF-12 rely on self-reported symptoms, which may be associated with inherent biases, such as desirability biases. However, these are widely validated and used tools, which ensure comparability between studies and settings.

## Conclusions

Our study highlights the impact of TB on mental and physical health-related QoL during and beyond treatment in the Southern African region with a high TB and HIV burden. We reported impairments at both mental and physical health levels at TB treatment start, with marked improvements during treatment and further improvements in some physical outcomes post-treatment. Traditionally, TB programs have focussed on successfully treating the infectious disease and its symptoms to prevent mortality and avoid morbidity. Our study adds to the knowledge of the global burden of end- and post-treatment TB and highlights the importance of QoL monitoring and care, especially for vulnerable populations, in addition to appropriate infectious disease management focussed on treatment completion. As such, our study advocates for integrated mental and physical health care and rehabilitation along with routine clinical TB care [37] using a patient-centred approach.

## Supplementary Information

Additional file 1: Tables S1–S6. Table S1 Number of study participants and proportion of visits, missing visits, deceased patients, and pending visits during the study period (at the start, end of tuberculosis treatment, and 6 months post-tuberculosis treatment). Table S2 Summary of mental and physical health-related quality of life (QoL) scores by study visit (tuberculosis treatment start, end of treatment, 6 months post-treatment). Table S3 Estimation results for the change in the QoL *z*-scores by predictor and time. Table S4 Estimated associations of baseline QoL scores with death during treatment. Table S5 Estimated associations of baseline QoL scores with any non-fatal serious adverse events during treatment. Table S6 Estimated associations of baseline QoL scores with missing follow-up visit at the end of treatment. Figures S1–S10. Fig. S1 Pairwise correlations between quality of life (QoL) scores by study visit. Fig. S2 Estimated proportion (in %) of patients with depressive symptoms for different cut-off values of the PHQ-9 score. Fig. S3 Estimated proportion (in %) of patients with impaired QoL by country. Fig. S4 The proportion of patients with impaired mental and physical QoL by study visit. Fig. S5 Trajectories of patients with depressive symptoms at any study visit (start of tuberculosis treatment, end of treatment, and 6 months post-treatment). Fig. S6 Distribution of the continuous QoL scores study visit. Fig. S7 Estimated change in QoL outcomes between end versus start of tuberculosis treatment and 6 months post-tuberculosis versus end of tuberculosis treatment. Fig. S8 Sensitivity analysis: estimated change in QoL when including missing follow-up visits and deaths for which the QoL scores were imputed. Fig. S9 Simulation analysis: estimated change in QoL for patients with follow-up visits (blue) vs hypothetical change in QoL for patients with missing follow-up visits or deaths so that the net effect would be zero (red). Fig. S10 Estimated change in QoL *z*-score (median as dot, 95%-CrI as lines) per quantile of the QoL score distribution at the end of tuberculosis treatment by patient characteristic.

## Data Availability

Complete data for this study cannot be posted in a supplemental file or a public repository because of legal and ethical restrictions. The Principles of Collaboration of this multi-national consortium and the regulatory requirements of the different countries’ IRBs require the submission and approval of individual project concept sheets that describe the planned analyses. Specifically, while the data held by the IeDEA-SA consortium may be available to other investigators, the proposed use must be based on a concept note that is approved by the regional Steering Groups (contact: https://www.iedea-sa.org/contact-us/).

## Abbreviations

6MWT: 6-Minute Walk Test
ASSIST: Alcohol, Smoking and Substance Involvement Screening Test
BMI: Body mass index
CrIs: 95%-Credible intervals
IQR: Interquartile range
MDR: Multidrug-resistant
PHQ-9: Patient Health Questionnaire-9
Pr: Posterior probability in %
SF-12: Short Form Health Survey
SF12-MCS: Mental Component Score
SF12-PCS: Physical Component Score
SGRQ: St. George Respiratory Questionnaire
STST: Sit-to-Stand Test
TB: Tuberculosis
