# Supplementary material for: Mental, physical, and respiratory health in people with tuberculosis in Southern Africa: a multi-country cohort analysis
Source: BMC Med. 2025 Aug 20;23:485. doi: 10.1186/s12916-025-04321-6 (PMC12366195; doi:10.1186/s12916-025-04321-6)
Supplement: Supplementary file 1 — Additional file 1: Tables S1–S6. Table S1 Number of study participants and proportion of visits, missing visits, deceased patients, and pending visits during the study period (at the start, end of tuberculosis treatment, and 6 months post-tuberculosis treatment). Table S2 Summary of mental and physical health-related quality of life (QoL) scores by study visit (tuberculosis treatment start, end of treatment, 6 months post-treatment). Table S3 Estimation results for the change in the QoL z-scores by predictor and time. Table S4 Estimated associations of baseline QoL scores with death during treatment. Table S5 Estimated associations of baseline QoL scores with any non-fatal serious adverse events during treatment. Table S6 Estimated associations of baseline QoL scores with missing follow-up visit at the end of treatment. Figures S1–S10. Fig. S1 Pairwise correlations between quality of life (QoL) scores by study visit. Fig. S2 Estimated proportion (in %) of patients with depressive symptoms for different cut-off values of the PHQ-9 score. Fig. S3 Estimated proportion (in %) of patients with impaired QoL by country. Fig. S4 The proportion of patients with impaired mental and physical QoL by study visit. Fig. S5 Trajectories of patients with depressive symptoms at any study visit (start of tuberculosis treatment, end of treatment, and 6 months post-treatment). Fig. S6 Distribution of the continuous QoL scores study visit. Fig. S7 Estimated change in QoL outcomes between end versus start of tuberculosis treatment and 6 months post-tuberculosis versus end of tuberculosis treatment. Fig. S8 Sensitivity analysis: estimated change in QoL when including missing follow-up visits and deaths for which the QoL scores were imputed. Fig. S9 Simulation analysis: estimated change in QoL for patients with follow-up visits (blue) vs hypothetical change in QoL for patients with missing follow-up visits or deaths so that the net effect would be zero (red). Fig. S10 Estimated change in Qo [file 12916_2025_4321_MOESM1_ESM.docx]

**ADDITIONAL FILE 1**

**Table S1. Number of study participants and proportion of visits, missing visits, deceased patients, and pending visits during the study period (at the start, end of tuberculosis treatment, and 6 months post-tuberculosis treatment).**

| **Study visit**  **(time point)** | **Visit** | **Missing visit** | **Deceased** | **Pending visit** |
| --- | --- | --- | --- | --- |
| Treatment start | 1,438 (100) | 0 (0) | 0 (0) | 0 (0) |
| Treatment end | 1,164 (81) | 148 (10) | 84 (6) | 42 (3) |
| Post-treatment | 745 (52) | 339 (24) | 99 (7) | 255 (18) |

**Table S2. Summary of mental and physical health-related Quality of Life (QoL) scores by study visit (tuberculosis treatment start, end of treatment, 6 months post-treatment).**

| **QoL outcome** |  | **Scores, median (IQR)** | | |
| --- | --- | --- | --- | --- |
|  |  | Treatment start | Treatment end | Post-treatment |
| Symptoms of depression (PHQ-9) |  | 6 (4-9) | 0 (0-2) | 0 (0-2) |
| Mental health (SF12-MCS) |  | 44 (38-50) | 53 (49-60) | 55 (50-62) |
| Physical health (SF12-PCS) |  | 35 (27-42) | 52 (46-56) | 54 (50-56) |
| Physical fitness (6MWT) |  | 360 (294-425) | 400 (324-471) | 420 (360-473) |
| Physical fitness (STST) |  | 17 (14-20) | 19 (17-22) | 20 (17-23) |
| Respiratory health (SGRQ) |  | 39 (27-52) | 11 (11-18) | 11 (11-12) |

6MWT, Six-Minute Walk Test; PHQ-9, Patient Health Questionnnaire-9; SF-12, Short Form Health Survey: Mental Component Score (SF12-MCS), Physical Component Score (SF12-PCS); SGRQ, St. George Respiratory Questionnaire; STST, Sit-to-Stand Test

IQR, interquartile range

**Table S3. Estimation results for the change in the QoL z-scores by predictor and time.** Median and 95%- Credibility Interval (CrI) of the association and the posterior probability that the change in QoL score “dz” is less or greater than zero are shown. PHQ-9 and SGRQ scores were transformed so that for all outcomes, a positive value indicates a positive association. Start: start of tuberculosis treatment, end: end of treatment, post: 6 months post-treatment.

| **Predictor** | **Outcome** | **Time** | **Median** | **Lower** | **Upper** | **Prob. dz < 0** | **Prob. dz > 0** |
| --- | --- | --- | --- | --- | --- | --- | --- |
| Age<30 | PHQ-9 | Start | 0.01 | -0.08 | 0.11 | 38 | 62 |
| Age<30 | PHQ-9 | End | 0.07 | -0.03 | 0.18 | 9 | 91 |
| Age<30 | PHQ-9 | Post | 0.08 | -0.05 | 0.21 | 12 | 88 |
| Age<30 | SF12-MCS | Start | 0.08 | -0.03 | 0.18 | 7 | 93 |
| Age<30 | SF12-MCS | End | 0.04 | -0.08 | 0.16 | 26 | 74 |
| Age<30 | SF12-MCS | Post | 0.10 | -0.04 | 0.25 | 9 | 91 |
| Age<30 | SF12-PCS | Start | 0.02 | -0.06 | 0.10 | 30 | 70 |
| Age<30 | SF12-PCS | End | 0.22 | 0.13 | 0.31 | 0 | 100 |
| Age<30 | SF12-PCS | Post | 0.13 | 0.02 | 0.24 | 1 | 99 |
| Age<30 | 6MWT | Start | 0.01 | -0.10 | 0.13 | 40 | 60 |
| Age<30 | 6MWT | End | 0.22 | 0.09 | 0.35 | 0 | 100 |
| Age<30 | 6MWT | Post | -0.04 | -0.18 | 0.10 | 71 | 29 |
| Age<30 | STST | Start | 0.18 | 0.04 | 0.31 | 1 | 99 |
| Age<30 | STST | End | 0.25 | 0.10 | 0.43 | 0 | 100 |
| Age<30 | STST | Post | 0.11 | -0.05 | 0.28 | 9 | 91 |
| Age<30 | SGRQ | Start | 0.05 | -0.04 | 0.13 | 13 | 87 |
| Age<30 | SGRQ | End | 0.09 | 0.01 | 0.18 | 2 | 98 |
| Age<30 | SGRQ | Post | 0.01 | -0.10 | 0.12 | 45 | 55 |
| Female | PHQ-9 | Start | -0.20 | -0.30 | -0.10 | 100 | 0 |
| Female | PHQ-9 | End | -0.07 | -0.18 | 0.03 | 91 | 9 |
| Female | PHQ-9 | Post | -0.06 | -0.19 | 0.07 | 83 | 17 |
| Female | SF12-MCS | Start | -0.11 | -0.21 | 0.00 | 98 | 2 |
| Female | SF12-MCS | End | 0.00 | -0.12 | 0.12 | 51 | 49 |
| Female | SF12-MCS | Post | -0.01 | -0.15 | 0.13 | 55 | 45 |
| Female | SF12-PCS | Start | -0.03 | -0.11 | 0.05 | 75 | 25 |
| Female | SF12-PCS | End | -0.15 | -0.24 | -0.05 | 100 | 0 |
| Female | SF12-PCS | Post | -0.08 | -0.19 | 0.03 | 91 | 9 |
| Female | 6MWT | Start | -0.39 | -0.52 | -0.24 | 100 | 0 |
| Female | 6MWT | End | -0.27 | -0.40 | -0.14 | 100 | 0 |
| Female | 6MWT | Post | -0.17 | -0.33 | -0.01 | 98 | 2 |
| Female | STST | Start | -0.22 | -0.36 | -0.07 | 100 | 0 |
| Female | STST | End | -0.09 | -0.24 | 0.06 | 89 | 11 |
| Female | STST | Post | -0.10 | -0.28 | 0.08 | 87 | 13 |
| Female | SGRQ | Start | -0.13 | -0.22 | -0.05 | 100 | 0 |
| Female | SGRQ | End | -0.07 | -0.16 | 0.02 | 93 | 7 |
| Female | SGRQ | Post | 0.02 | -0.09 | 0.13 | 39 | 61 |
| HIV+ | PHQ-9 | Start | -0.05 | -0.14 | 0.04 | 86 | 14 |
| HIV+ | PHQ-9 | End | -0.07 | -0.17 | 0.03 | 91 | 9 |
| HIV+ | PHQ-9 | Post | 0.01 | -0.12 | 0.13 | 47 | 53 |
| HIV+ | SF12-MCS | Start | -0.06 | -0.16 | 0.03 | 89 | 11 |
| HIV+ | SF12-MCS | End | -0.10 | -0.21 | 0.01 | 96 | 4 |
| HIV+ | SF12-MCS | Post | 0.02 | -0.12 | 0.15 | 40 | 60 |
| HIV+ | SF12-PCS | Start | -0.13 | -0.20 | -0.05 | 100 | 0 |
| HIV+ | SF12-PCS | End | 0.05 | -0.03 | 0.14 | 11 | 89 |
| HIV+ | SF12-PCS | Post | 0.07 | -0.03 | 0.18 | 8 | 92 |
| HIV+ | 6MWT | Start | -0.06 | -0.19 | 0.04 | 87 | 13 |
| HIV+ | 6MWT | End | -0.07 | -0.18 | 0.06 | 85 | 15 |
| HIV+ | 6MWT | Post | -0.03 | -0.16 | 0.11 | 66 | 34 |
| HIV+ | STST | Start | -0.03 | -0.16 | 0.09 | 71 | 29 |
| HIV+ | STST | End | -0.03 | -0.17 | 0.12 | 67 | 33 |
| HIV+ | STST | Post | 0.02 | -0.13 | 0.18 | 38 | 62 |
| HIV+ | SGRQ | Start | -0.05 | -0.13 | 0.02 | 92 | 8 |
| HIV+ | SGRQ | End | 0.01 | -0.07 | 0.10 | 38 | 62 |
| HIV+ | SGRQ | Post | 0.01 | -0.10 | 0.11 | 43 | 57 |
| MDR | PHQ-9 | Start | -0.07 | -0.30 | 0.17 | 71 | 29 |
| MDR | PHQ-9 | End | 0.23 | -0.06 | 0.52 | 6 | 94 |
| MDR | PHQ-9 | Post | -0.18 | -0.72 | 0.37 | 74 | 26 |
| MDR | SF12-MCS | Start | -0.12 | -0.36 | 0.12 | 84 | 16 |
| MDR | SF12-MCS | End | 0.36 | 0.00 | 0.70 | 3 | 97 |
| MDR | SF12-MCS | Post | -0.06 | -0.66 | 0.55 | 58 | 42 |
| MDR | SF12-PCS | Start | 0.00 | -0.19 | 0.19 | 50 | 50 |
| MDR | SF12-PCS | End | 0.02 | -0.25 | 0.29 | 43 | 57 |
| MDR | SF12-PCS | Post | 0.26 | -0.21 | 0.74 | 14 | 86 |
| MDR | 6MWT | Start | -0.22 | -0.50 | 0.06 | 94 | 6 |
| MDR | 6MWT | End | -0.09 | -0.43 | 0.28 | 68 | 32 |
| MDR | 6MWT | Post | -0.11 | -0.67 | 0.43 | 65 | 35 |
| MDR | STST | Start | -0.08 | -0.41 | 0.23 | 69 | 31 |
| MDR | STST | End | 0.38 | -0.04 | 0.79 | 4 | 96 |
| MDR | STST | Post | -0.05 | -0.67 | 0.57 | 56 | 44 |
| MDR | SGRQ | Start | -0.15 | -0.34 | 0.03 | 95 | 5 |
| MDR | SGRQ | End | 0.01 | -0.22 | 0.25 | 46 | 54 |
| MDR | SGRQ | Post | -0.14 | -0.60 | 0.32 | 72 | 28 |
| History of TB | PHQ-9 | Start | -0.14 | -0.25 | -0.03 | 99 | 1 |
| History of TB | PHQ-9 | End | -0.18 | -0.31 | -0.06 | 100 | 0 |
| History of TB | PHQ-9 | Post | -0.08 | -0.24 | 0.07 | 86 | 14 |
| History of TB | SF12-MCS | Start | -0.04 | -0.17 | 0.08 | 76 | 24 |
| History of TB | SF12-MCS | End | -0.25 | -0.39 | -0.11 | 100 | 0 |
| History of TB | SF12-MCS | Post | -0.24 | -0.41 | -0.07 | 100 | 0 |
| History of TB | SF12-PCS | Start | -0.07 | -0.17 | 0.02 | 93 | 7 |
| History of TB | SF12-PCS | End | -0.12 | -0.23 | -0.01 | 98 | 2 |
| History of TB | SF12-PCS | Post | -0.23 | -0.36 | -0.09 | 100 | 0 |
| History of TB | 6MWT | Start | -0.06 | -0.18 | 0.06 | 82 | 18 |
| History of TB | 6MWT | End | -0.07 | -0.21 | 0.08 | 80 | 20 |
| History of TB | 6MWT | Post | -0.12 | -0.28 | 0.05 | 92 | 8 |
| History of TB | STST | Start | 0.05 | -0.10 | 0.20 | 25 | 75 |
| History of TB | STST | End | -0.09 | -0.27 | 0.07 | 87 | 13 |
| History of TB | STST | Post | -0.13 | -0.32 | 0.05 | 92 | 8 |
| History of TB | SGRQ | Start | -0.09 | -0.18 | 0.00 | 97 | 3 |
| History of TB | SGRQ | End | -0.11 | -0.22 | -0.01 | 98 | 2 |
| History of TB | SGRQ | Post | -0.18 | -0.31 | -0.05 | 100 | 0 |
| Drinking | PHQ-9 | Start | -0.06 | -0.19 | 0.10 | 77 | 23 |
| Drinking | PHQ-9 | End | 0.00 | -0.14 | 0.13 | 51 | 49 |
| Drinking | PHQ-9 | Post | 0.01 | -0.15 | 0.16 | 46 | 54 |
| Drinking | SF12-MCS | Start | -0.17 | -0.32 | 0.00 | 97 | 3 |
| Drinking | SF12-MCS | End | -0.01 | -0.17 | 0.13 | 58 | 42 |
| Drinking | SF12-MCS | Post | -0.08 | -0.26 | 0.11 | 79 | 21 |
| Drinking | SF12-PCS | Start | -0.03 | -0.15 | 0.09 | 68 | 32 |
| Drinking | SF12-PCS | End | 0.08 | -0.04 | 0.22 | 10 | 90 |
| Drinking | SF12-PCS | Post | 0.07 | -0.08 | 0.21 | 17 | 83 |
| Drinking | 6MWT | Start | -0.12 | -0.24 | 0.02 | 95 | 5 |
| Drinking | 6MWT | End | -0.01 | -0.17 | 0.15 | 54 | 46 |
| Drinking | 6MWT | Post | 0.04 | -0.13 | 0.22 | 32 | 68 |
| Drinking | STST | Start | -0.21 | -0.36 | -0.05 | 100 | 0 |
| Drinking | STST | End | -0.07 | -0.26 | 0.14 | 72 | 28 |
| Drinking | STST | Post | 0.17 | -0.05 | 0.41 | 7 | 93 |
| Drinking | SGRQ | Start | -0.07 | -0.19 | 0.06 | 87 | 13 |
| Drinking | SGRQ | End | 0.05 | -0.07 | 0.16 | 20 | 80 |
| Drinking | SGRQ | Post | 0.08 | -0.06 | 0.22 | 13 | 87 |
| Smoking | PHQ-9 | Start | -0.18 | -0.31 | -0.05 | 100 | 0 |
| Smoking | PHQ-9 | End | -0.04 | -0.18 | 0.09 | 74 | 26 |
| Smoking | PHQ-9 | Post | -0.11 | -0.25 | 0.04 | 93 | 7 |
| Smoking | SF12-MCS | Start | -0.01 | -0.15 | 0.14 | 54 | 46 |
| Smoking | SF12-MCS | End | -0.06 | -0.21 | 0.09 | 77 | 23 |
| Smoking | SF12-MCS | Post | 0.02 | -0.15 | 0.19 | 41 | 59 |
| Smoking | SF12-PCS | Start | -0.07 | -0.20 | 0.05 | 87 | 13 |
| Smoking | SF12-PCS | End | -0.01 | -0.13 | 0.10 | 57 | 43 |
| Smoking | SF12-PCS | Post | -0.08 | -0.22 | 0.05 | 88 | 12 |
| Smoking | 6MWT | Start | 0.03 | -0.13 | 0.15 | 36 | 64 |
| Smoking | 6MWT | End | 0.02 | -0.13 | 0.20 | 39 | 61 |
| Smoking | 6MWT | Post | 0.01 | -0.19 | 0.19 | 47 | 53 |
| Smoking | STST | Start | 0.09 | -0.10 | 0.25 | 18 | 82 |
| Smoking | STST | End | 0.08 | -0.12 | 0.28 | 24 | 76 |
| Smoking | STST | Post | -0.21 | -0.42 | 0.06 | 93 | 7 |
| Smoking | SGRQ | Start | -0.18 | -0.32 | -0.06 | 100 | 0 |
| Smoking | SGRQ | End | 0.01 | -0.10 | 0.12 | 41 | 59 |
| Smoking | SGRQ | Post | 0.02 | -0.11 | 0.14 | 39 | 61 |

6MWT, Six-Minute Walk Test; PHQ-9, Patient Health Questionnnaire-9; SF-12, Short Form Health Survey: Mental Component Score (SF12-MCS), Physical Component Score (SF12-PCS); SGRQ, St. George Respiratory Questionnaire; STST, Sit-to-Stand Test

**Table S4. Estimated associations of baseline QoL scores with death during treatment.** Median and 95%-Credibility Interval (CrI) of the change in the QoL z-score. The PHQ-9 and SGRQ scores were transformed so that for all outcomes, a positive change indicates an improvement.

| **QoL outcome** |  | **Change in QoL z-scores, posterior probability** | | | |
| --- | --- | --- | --- | --- | --- |
|  |  | **Median** | **Lower** | **Upper** |  |
| Symptoms of depression (PHQ-9) |  | -0.56 | -0.78 | -0.34 |  |
| Mental health (SF12-MCS) |  | -0.45 | -0.67 | -0.22 |  |
| Physical health (SF12-PCS) |  | -0.48 | -0.69 | -0.27 |  |
| Physical fitness (6MWT) |  | -0.32 | -0.54 | -0.09 |  |
| Physical fitness (STST) |  | -0.32 | -0.60 | -0.03 |  |
| Respiratory health (SGRQ) |  | -0.65 | -0.87 | -0.45 |  |

6MWT, Six-Minute Walk Test; PHQ-9, Patient Health Questionnnaire-9; SF-12, Short Form Health Survey: Mental Component Score (SF12-MCS), Physical Component Score (SF12-PCS); SGRQ, St. George Respiratory Questionnaire; STST, Sit-to-Stand Test

**Table S5. Estimated associations of baseline QoL scores with any non-fatal serious adverse events during treatment.** Median and 95%-Credibility Interval (CrI) of the change in the QoL z-score. The PHQ-9 and SGRQ scores were transformed so that for all outcomes, a positive change indicates an improvement.

| **QoL outcome** |  | **Change in QoL z-scores, posterior probability** | | |
| --- | --- | --- | --- | --- |
|  |  | **Median** | **Lower** | **Upper** |
| Symptoms of depression (PHQ-9) |  | 0.34 | 0.00 | 0.68 |
| Mental health (SF12-MCS) |  | -0.40 | -0.74 | -0.07 |
| Physical health (SF12-PCS) |  | -0.54 | -0.86 | -0.23 |
| Physical fitness (6MWT) |  | -0.48 | -0.81 | -0.13 |
| Physical fitness (STST) |  | 0.21 | -0.33 | 0.73 |
| Respiratory health (SGRQ) |  | -0.54 | -0.86 | -0.22 |

6MWT, Six-Minute Walk Test; PHQ-9, Patient Health Questionnnaire-9; SF-12, Short Form Health Survey: Mental Component Score (SF12-MCS), Physical Component Score (SF12-PCS); SGRQ, St. George Respiratory Questionnaire; STST, Sit-to-Stand Test

**Table S6. Estimated associations of baseline QoL scores with missing follow-up visit at the end of treatment.** Median and 95%-Credibility Interval (CrI) of the change in the QoL z-score. The PHQ-9 and SGRQ scores were transformed so that for all outcomes, a positive change indicates an improvement.

| **QoL outcome** |  | **Change in QoL z-scores, posterior probability** | | |
| --- | --- | --- | --- | --- |
|  |  | **Median** | **Lower** | **Upper** |
| Symptoms of depression (PHQ-9) |  | -0.06 | -0.23 | 0.11 |
| Mental health (SF12-MCS) |  | -0.08 | -0.25 | 0.10 |
| Physical health (SF12-PCS) |  | -0.05 | -0.21 | 0.11 |
| Physical fitness (6MWT) |  | -0.12 | -0.28 | 0.06 |
| Physical fitness (STST) |  | -0.06 | -0.25 | 0.13 |
| Respiratory health (SGRQ) |  | -0.06 | -0.22 | 0.10 |

6MWT, Six-Minute Walk Test; PHQ-9, Patient Health Questionnnaire-9; SF-12, Short Form Health Survey: Mental Component Score (SF12-MCS), Physical Component Score (SF12-PCS); SGRQ, St. George Respiratory Questionnaire; STST, Sit-to-Stand Test

**Figure S1. Pairwise correlations between Quality of Life (QoL) scores by study visit.** Stars indicate significance levels: p < 0.05 (*), p < 0.01 (**), p < 0.001 (***).


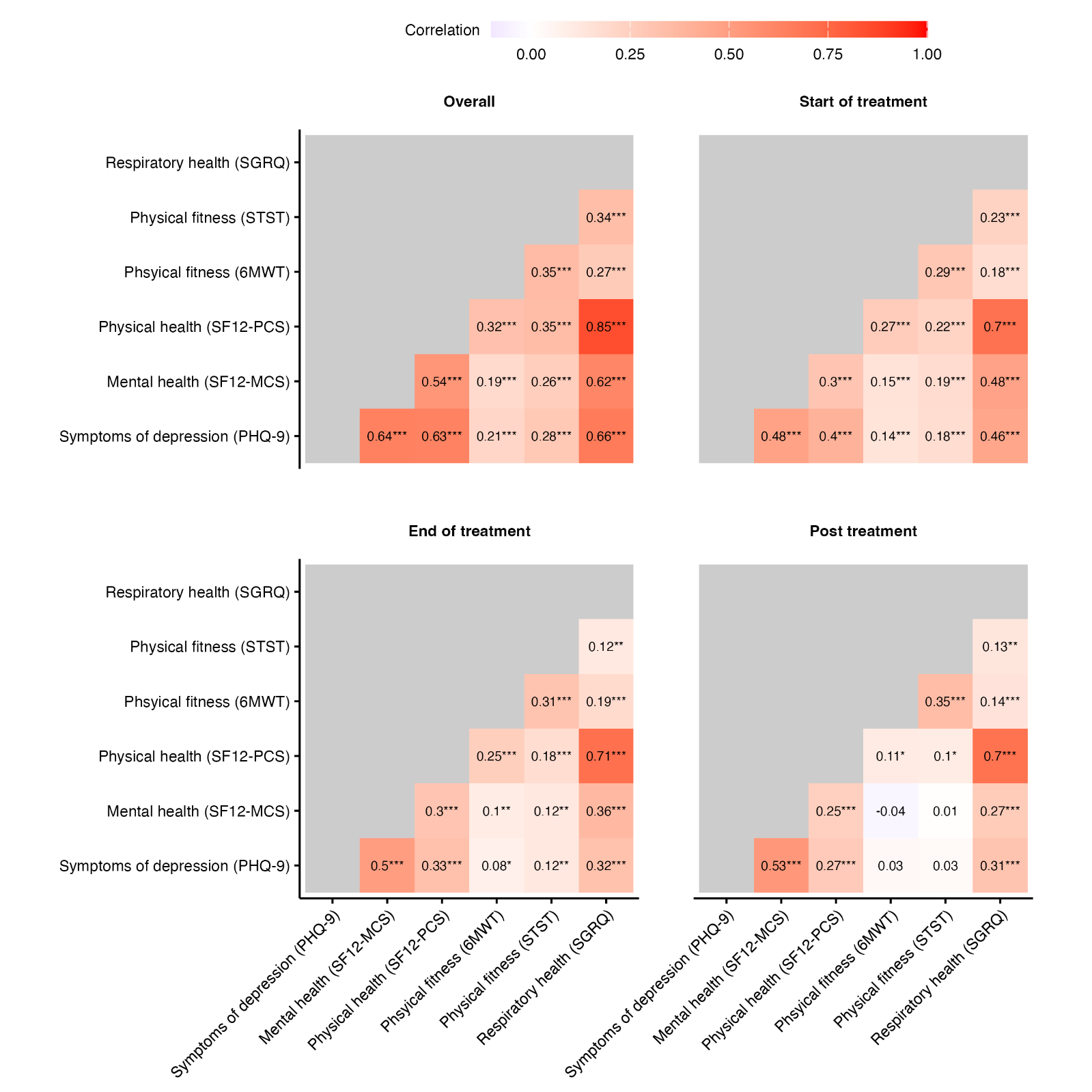


6MWT, Six-Minute Walk Test; PHQ-9, Patient Health Questionnnaire-9; SF-12, Short Form Health Survey: Mental Component Score (SF12-MCS), Physical Component Score (SF12-PCS); SGRQ, St. George Respiratory Questionnaire; STST, Sit-to-Stand Test

**Figure S2: Estimated proportion (in %) of patients with depressive symptoms for different cut-off values of the PHQ-9 score.** The region-specific cut-off of ≥7 was selected based on validation studies for Malawi, Mozambique, South Africa and Zimbabwe (23-26); the cut-off of ≥10 was used based on the original study (17,27). Bars show the mean proportion and errorbars the standard deviation and are annotated with the posterior probability that the proportion of patients with impaired QoL is smaller at the end versus the start of TB treatment (PES) and the probability that the proportion of patients with impaired QoL is smaller at 6 months post-treatment versus the end of treatment (PPE).

**
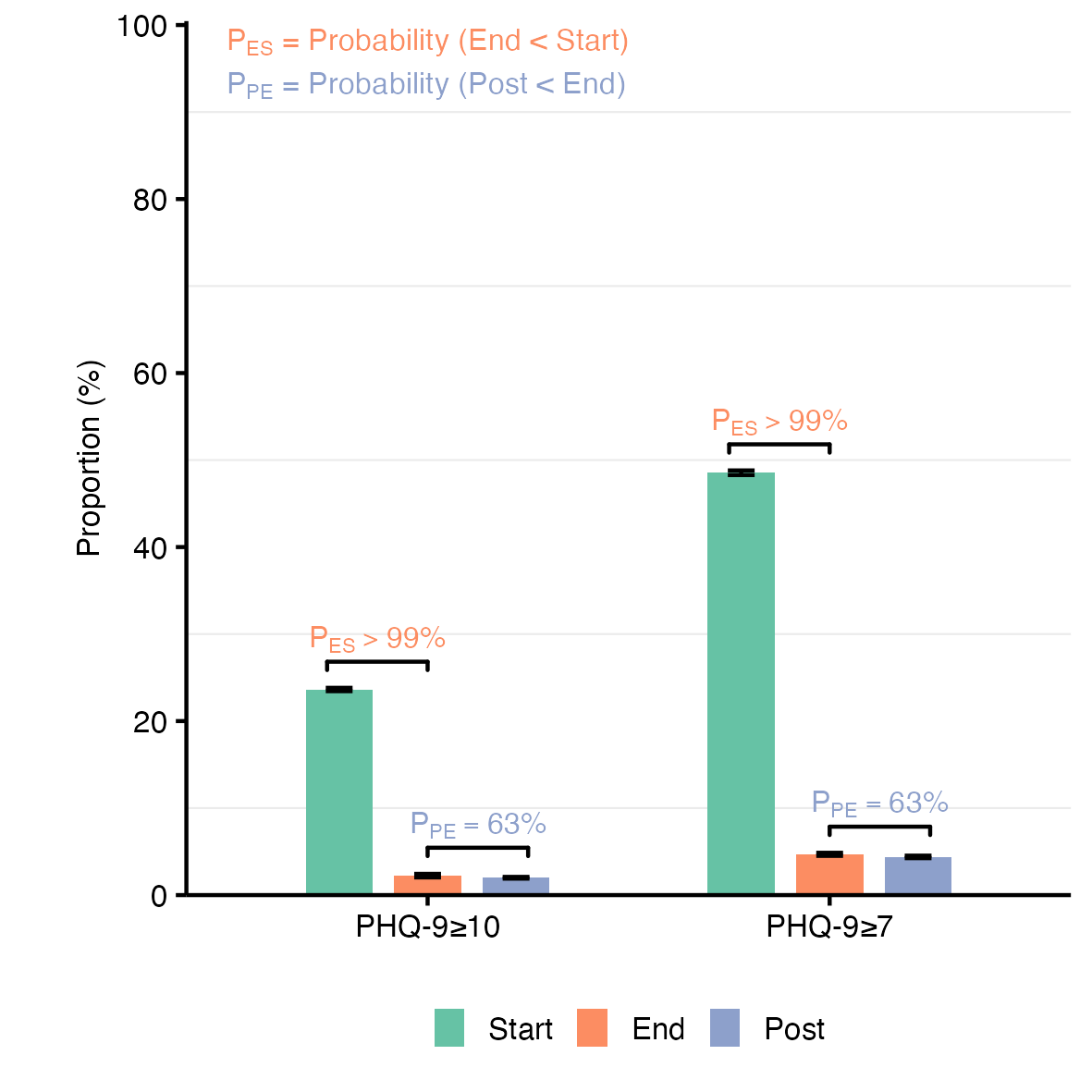
**

**Figure S3: Estimated proportion (in %) of patients with impaired QoL by country.** Bars show the mean proportion and errorbars the standard deviation and are annotated with the posterior probability that the proportion of patients with impaired QoL is smaller at the end versus the start of TB treatment (PES) and the probability that the proportion of patients with impaired QoL is smaller at 6 months post-treatment versus the end of treatment (PPE).


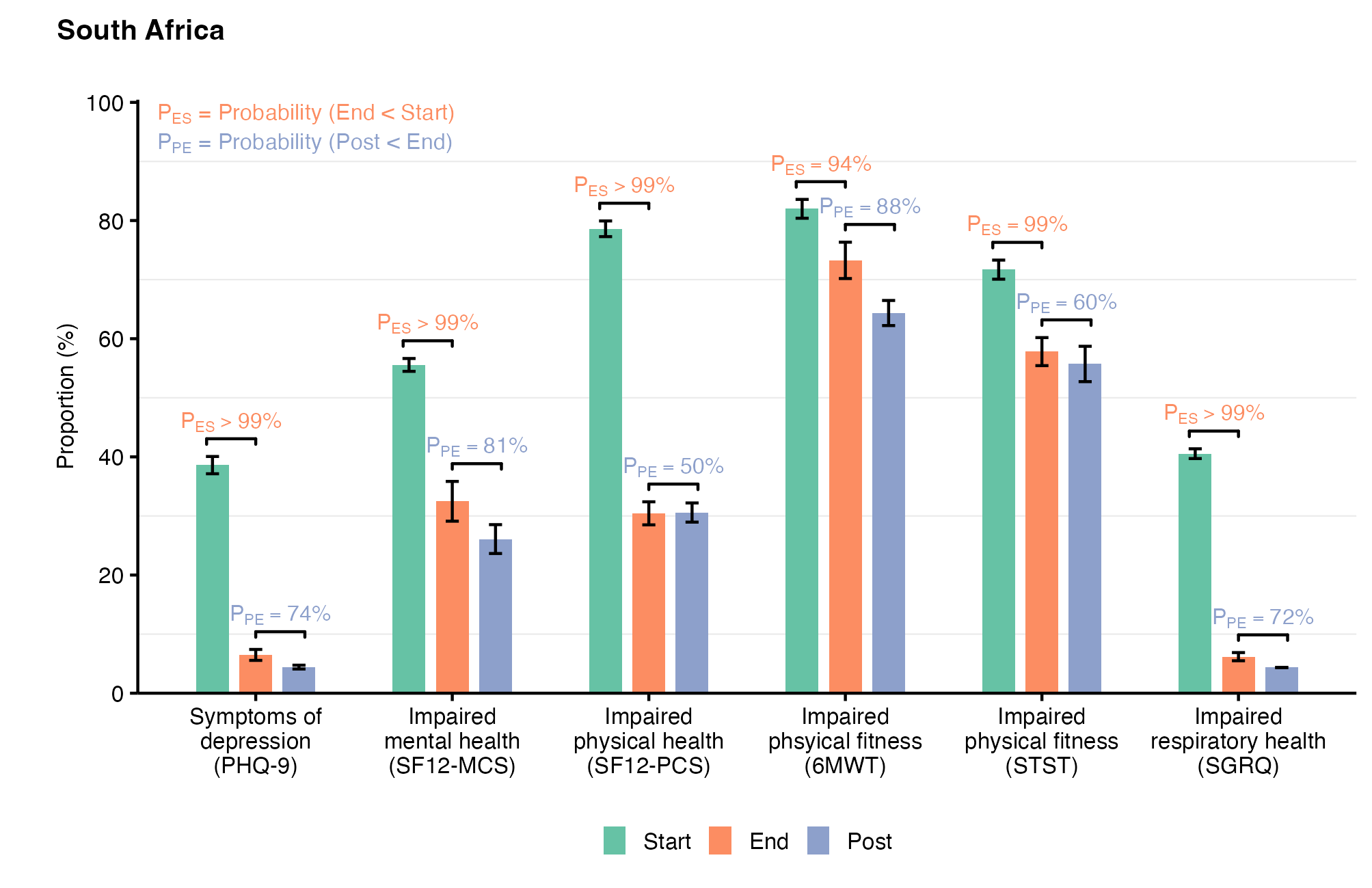

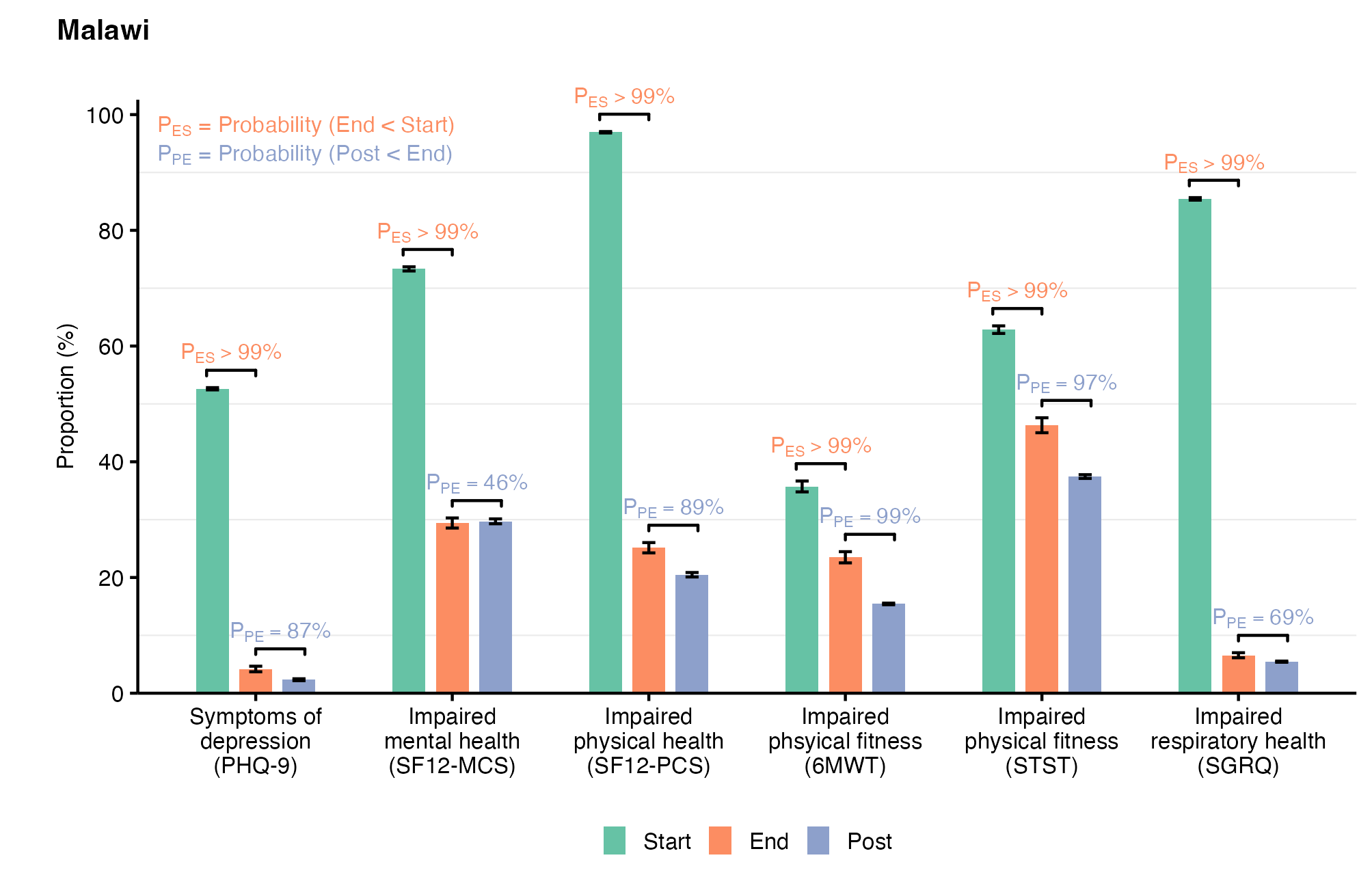

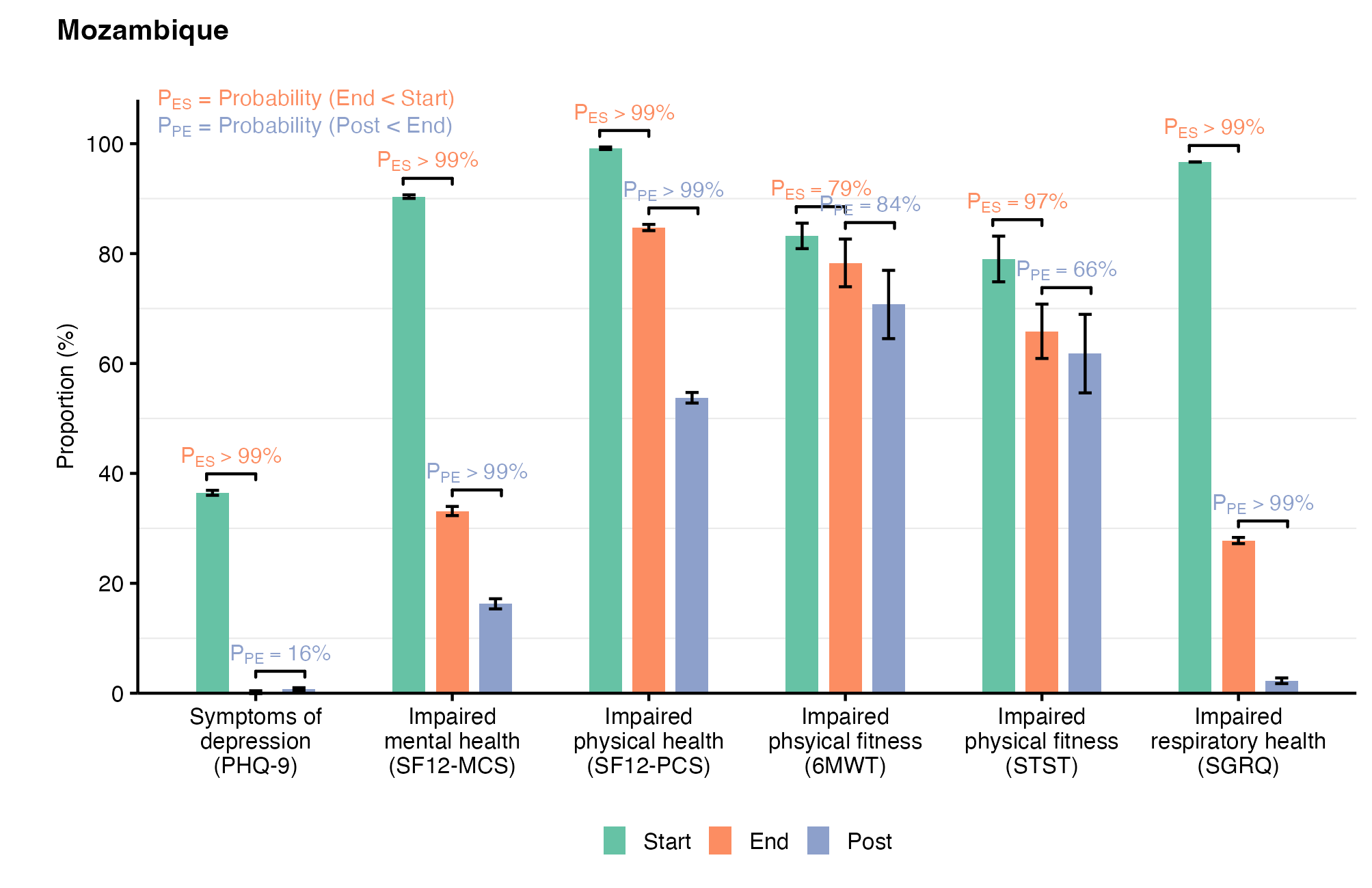

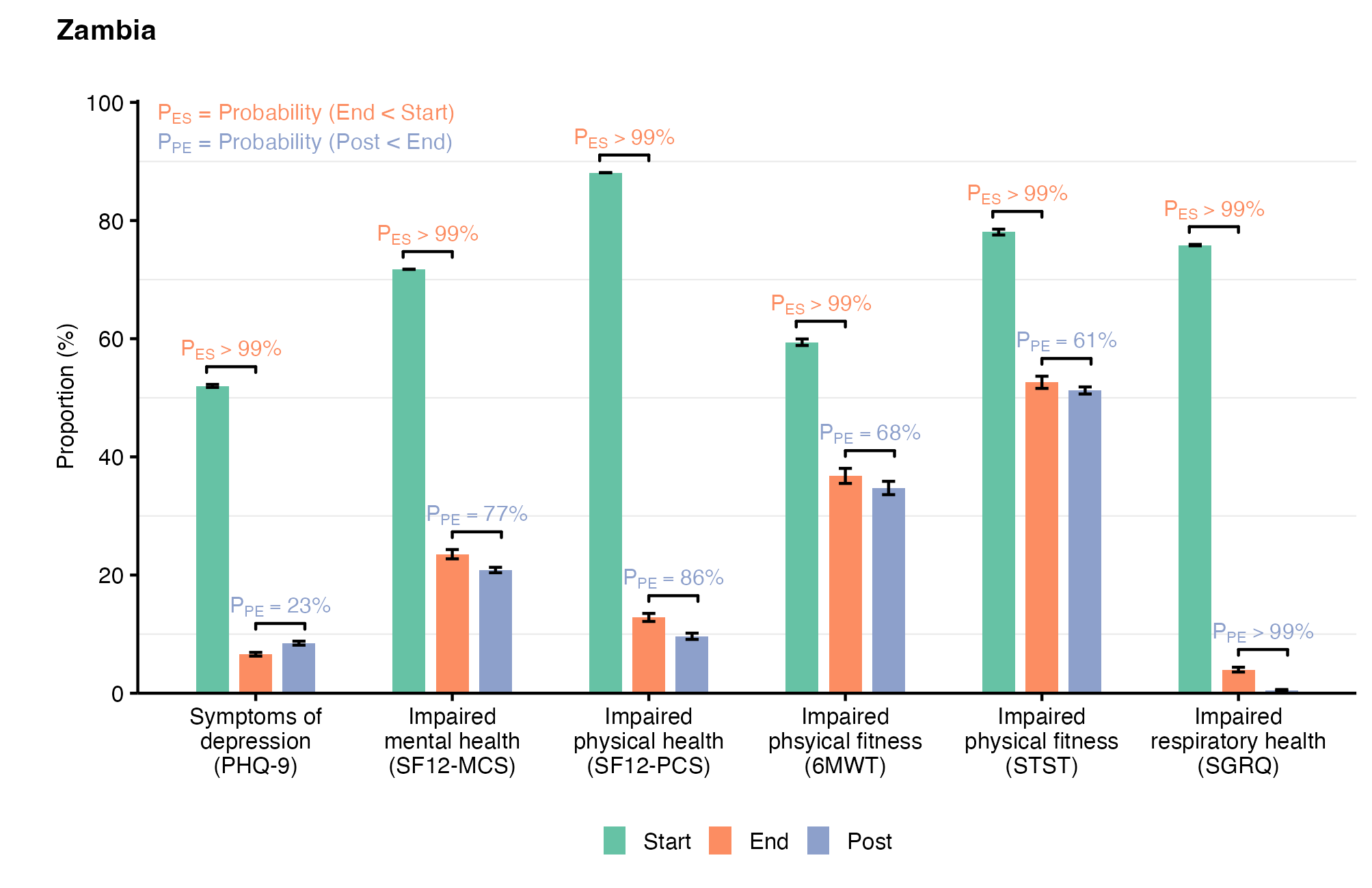

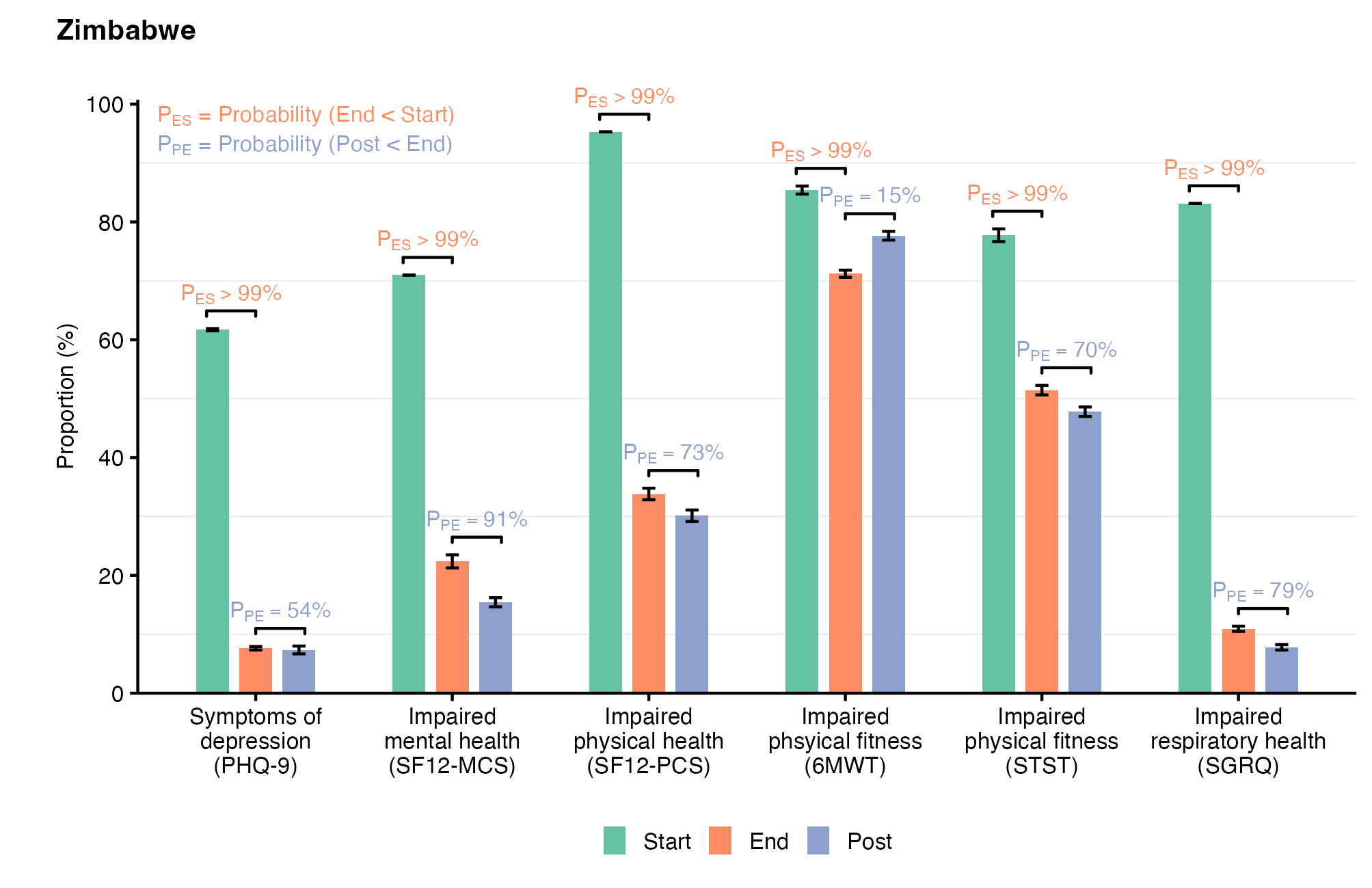


6MWT, Six-Minute Walk Test; PHQ-9, Patient Health Questionnnaire-9; SF-12, Short Form Health Survey: Mental Component Score (SF12-MCS), Physical Component Score (SF12-PCS); SGRQ, St. George Respiratory Questionnaire; STST, Sit-to-Stand Test

Note that the physical fitness scores and corresponding proportions for Mosambique were fully imputed.

**Figure S4. The proportion of patients with impaired mental and physical QoL by study visit.** Start: start of tuberculosis treatment, end: end of treatment, post: 6 months post-treatment.


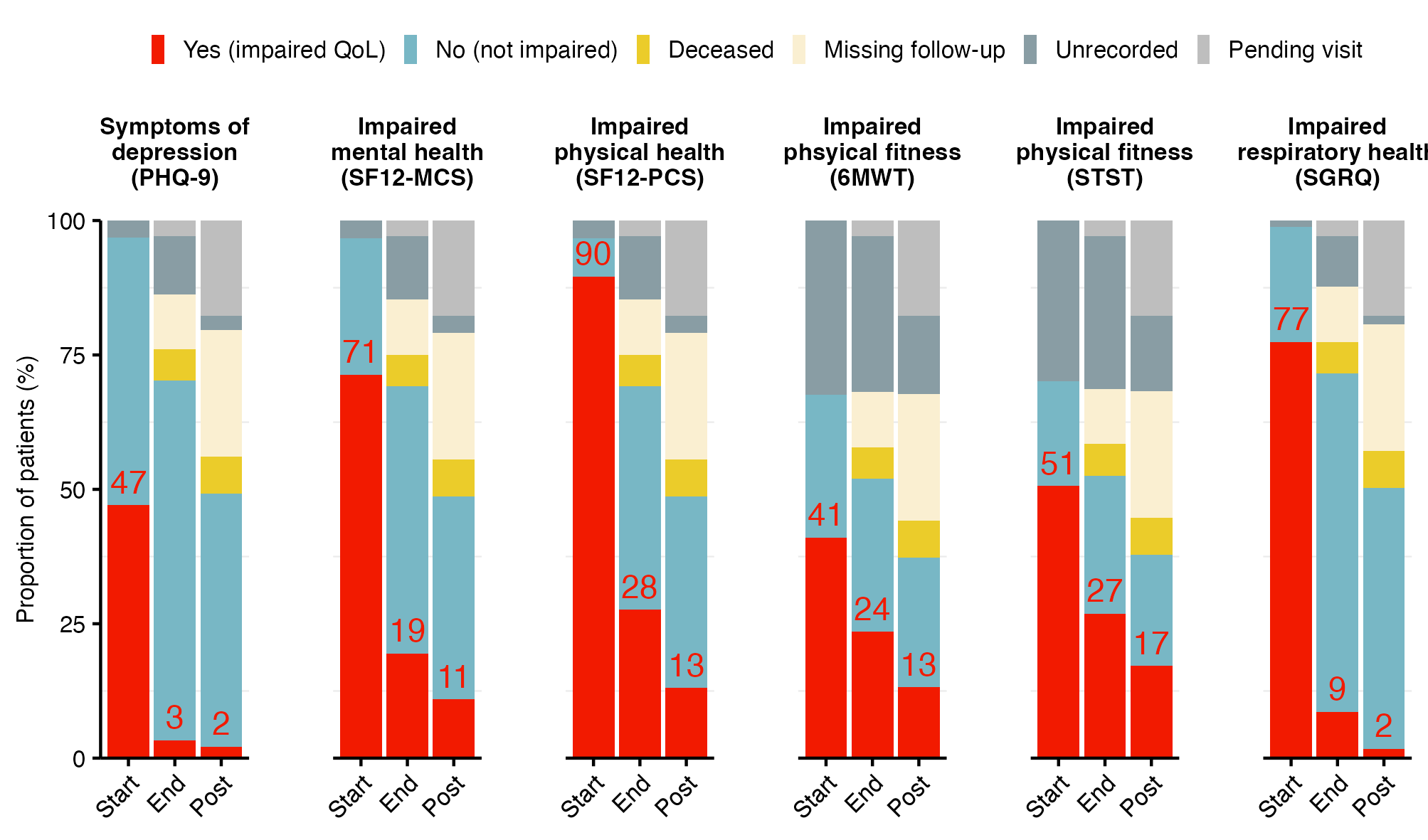


6MWT, Six-Minute Walk Test; PHQ-9, Patient Health Questionnnaire-9; SF-12, Short Form Health Survey: Mental Component Score (SF12-MCS), Physical Component Score (SF12-PCS); SGRQ, St. George Respiratory Questionnaire; STST, Sit-to-Stand Test

**Figure S5. Trajectories of patients with depressive symptoms at any study visit (start of tuberculosis treatment, end of treatment, and 6 months post-treatment).** Depressive symptoms are defined as PHQ-9 ≥7.


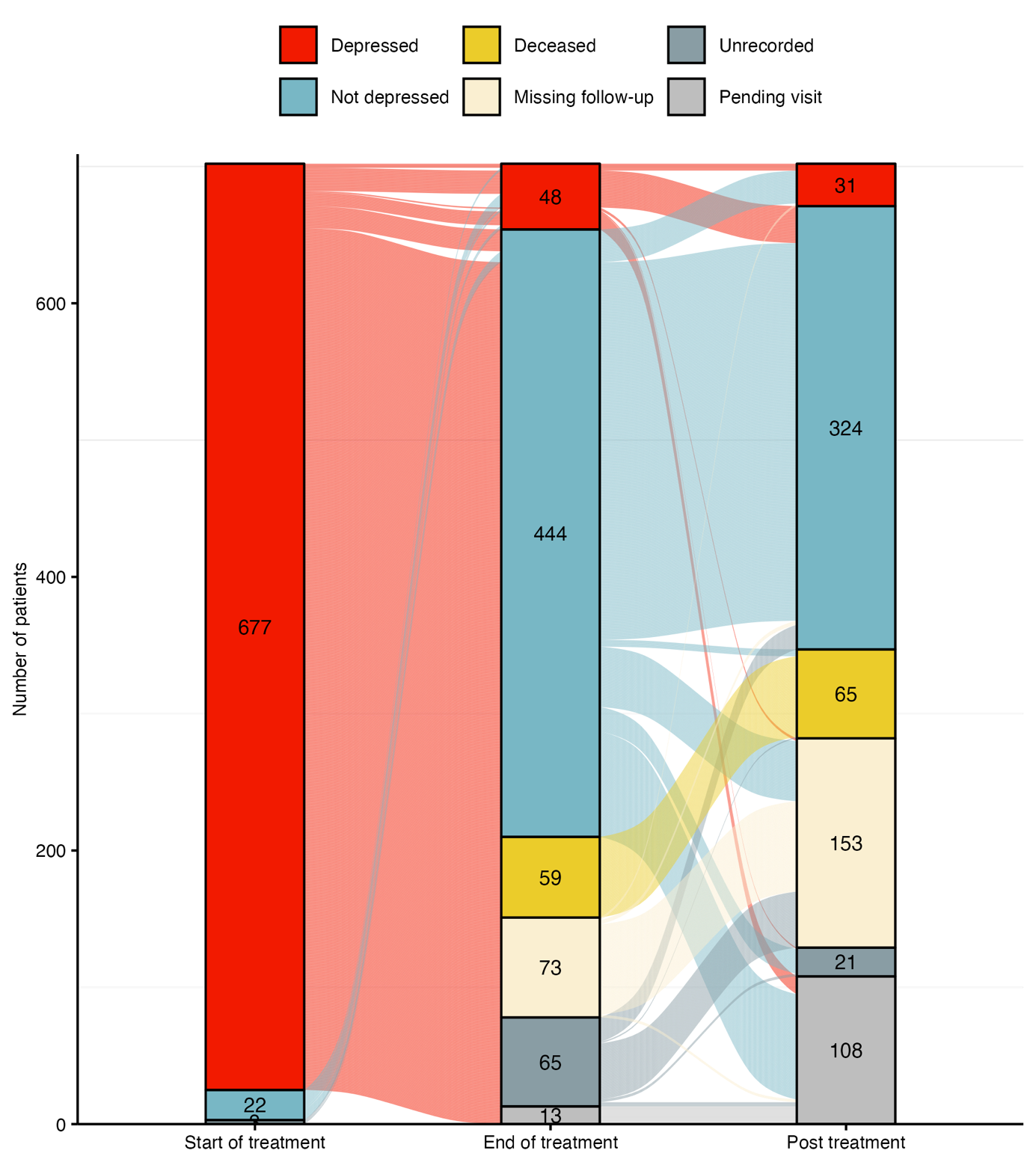


**Figure S6: Distribution of the continuous QoL scores study visit.** Boxplots show medians as lines, interquartile ranges (IQR) as boxes, 1.5 times IQR as whiskers, and outliers as dots. See Supplementary Table 2 for numerical results. Start: start of tuberculosis treatment, end: end of treatment, post: 6 months post-treatment.


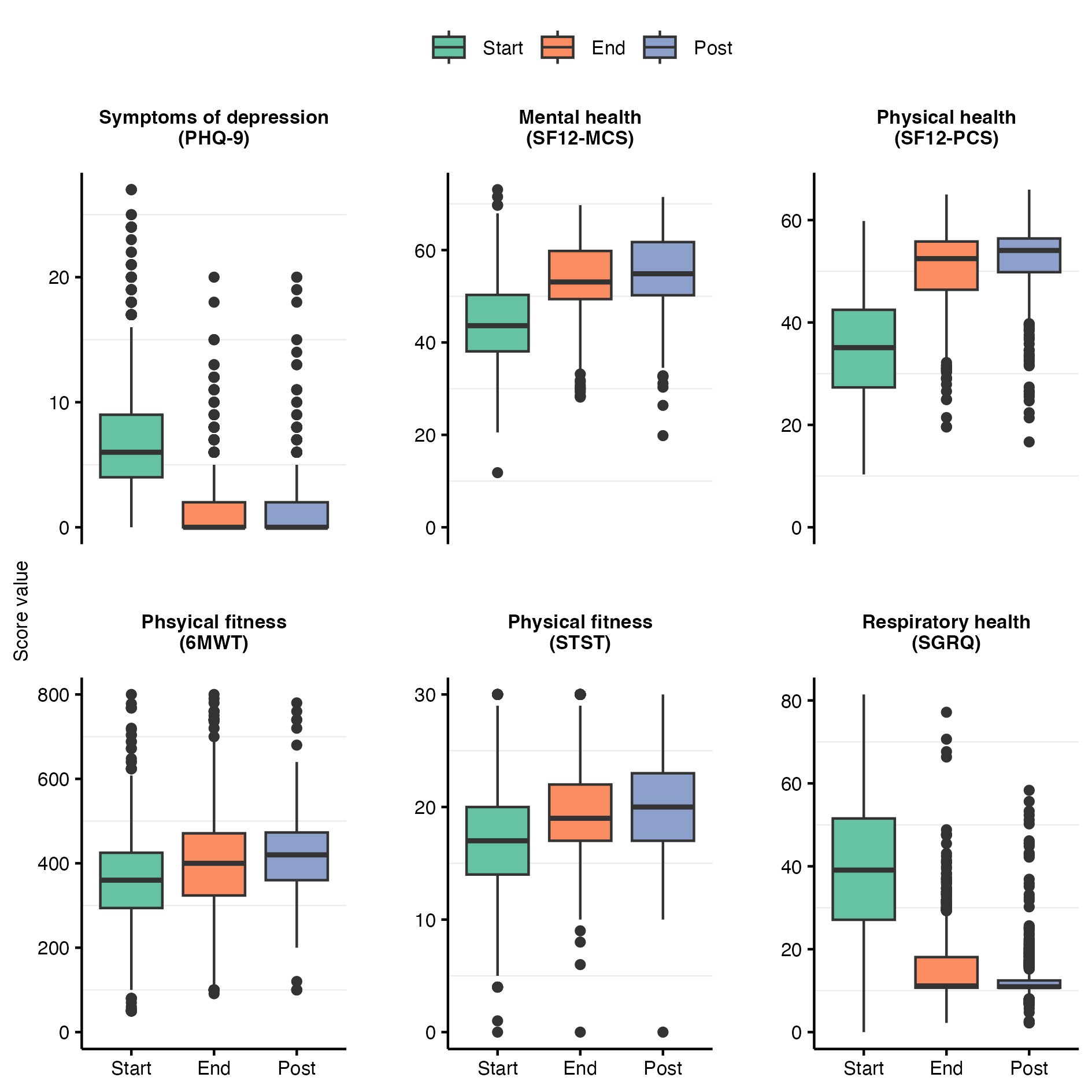


6MWT, Six-Minute Walk Test; PHQ-9, Patient Health Questionnnaire-9; SF-12, Short Form Health Survey: Mental Component Score (SF12-MCS), Physical Component Score (SF12-PCS); SGRQ, St. George Respiratory Questionnaire; STST, Sit-to-Stand Test

**Figure S7: Estimated change in QoL outcomes between end versus start of tuberculosis treatment and 6 months post-tuberculosis versus end of tuberculosis treatment.** PHQ-9 and SGRQ scores were transformed so that for all outcomes, a positive change means an improvement.


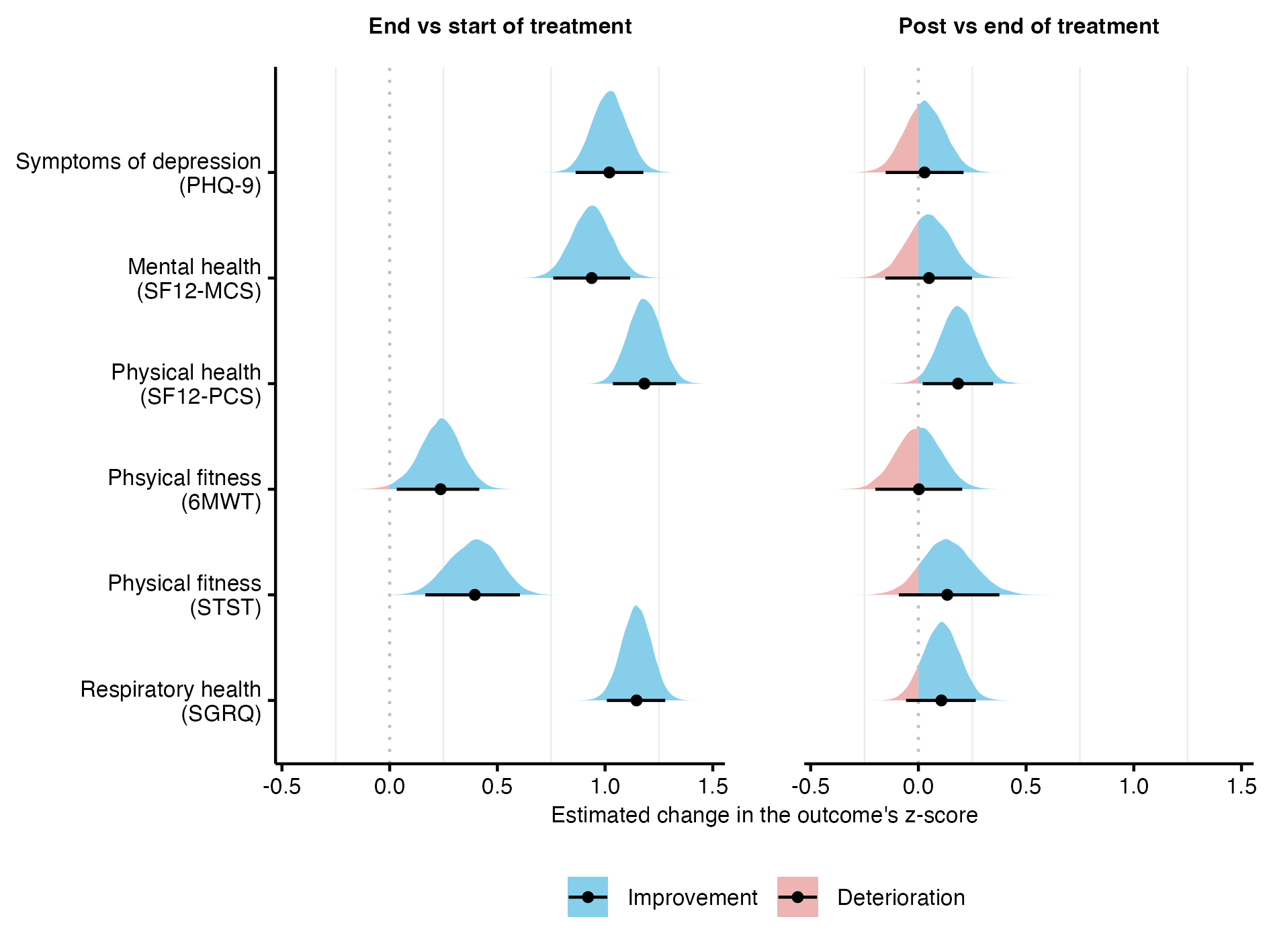


6MWT, Six-Minute Walk Test; PHQ-9, Patient Health Questionnnaire-9; SF-12, Short Form Health Survey: Mental Component Score (SF12-MCS), Physical Component Score (SF12-PCS); SGRQ, St. George Respiratory Questionnaire; STST, Sit-to-Stand Test

**Figure S8. Sensitivity analysis: Estimated change in QoL when including missing follow-up visits and deaths for which the QoL scores were imputed.** End versus the start of tuberculosis treatment and 6 months post-tuberculosis *versus* the end of tuberculosis treatment. PHQ-9 and SGRQ scores were transformed so that for all outcomes, a positive change indicates an improvement.

**
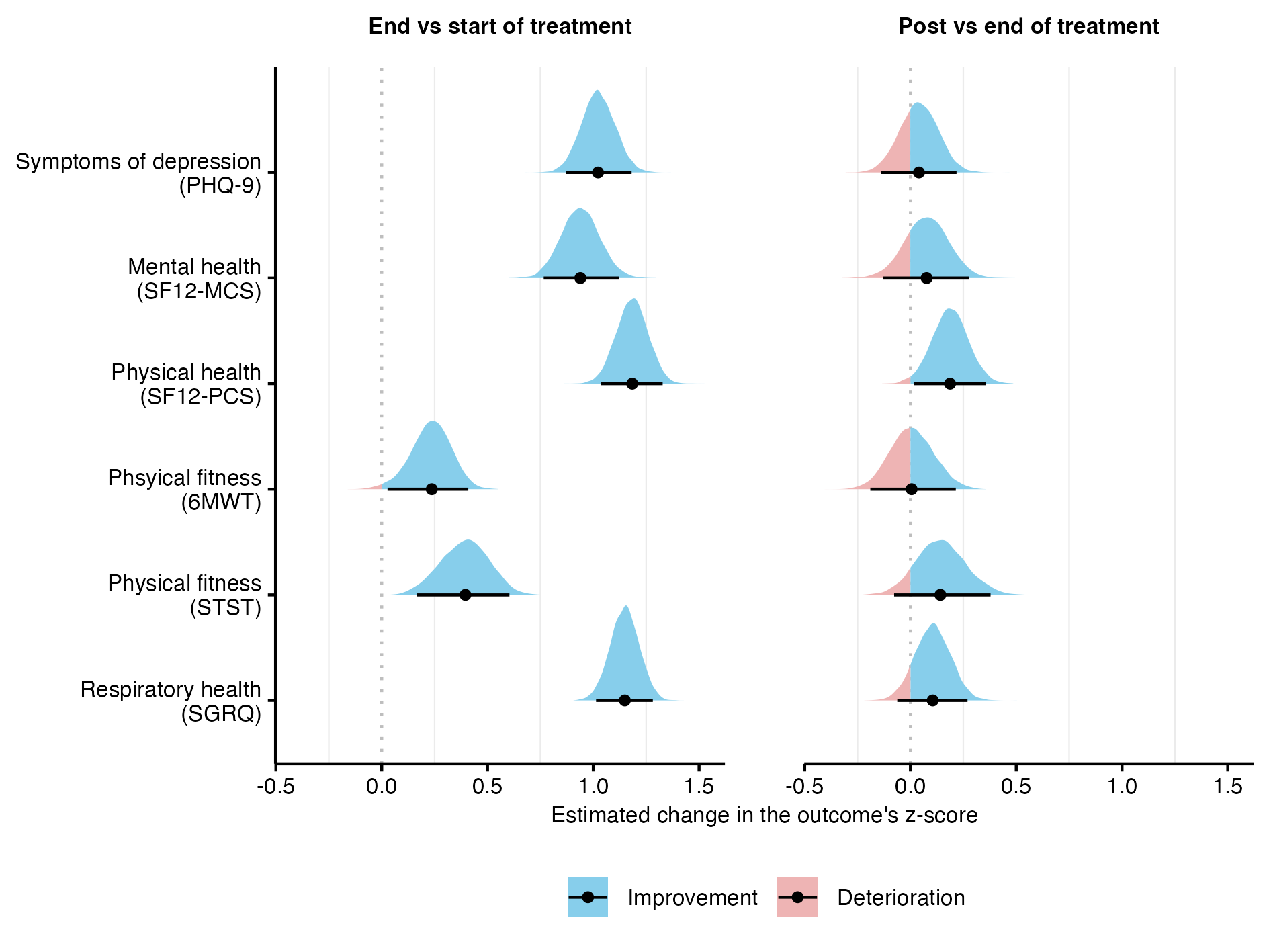
**

As a sensitivity analysis, we re-estimated changes in QoL scores including missing follow-ups and deaths with their fully imputed outcomes. If a patient died or did not return for a follow-up visit at the end of treatment, we only included the imputed outcomes at the end of tuberculosis treatment, excluding the post-treatment visit.

**Figure S9. Simulation analysis: Estimated change in QoL for patients with follow-up visits (blue) vs hypothetical change in QoL for patients with missing follow-up visits or deaths so that the net effect would be zero (red).** Hypothetical changes well below zero imply that significant decreases in QoL for deceased patients or patients with missing follow-up visits would be required to offset the observed increases in QoL for patients with follow-up visits.

**
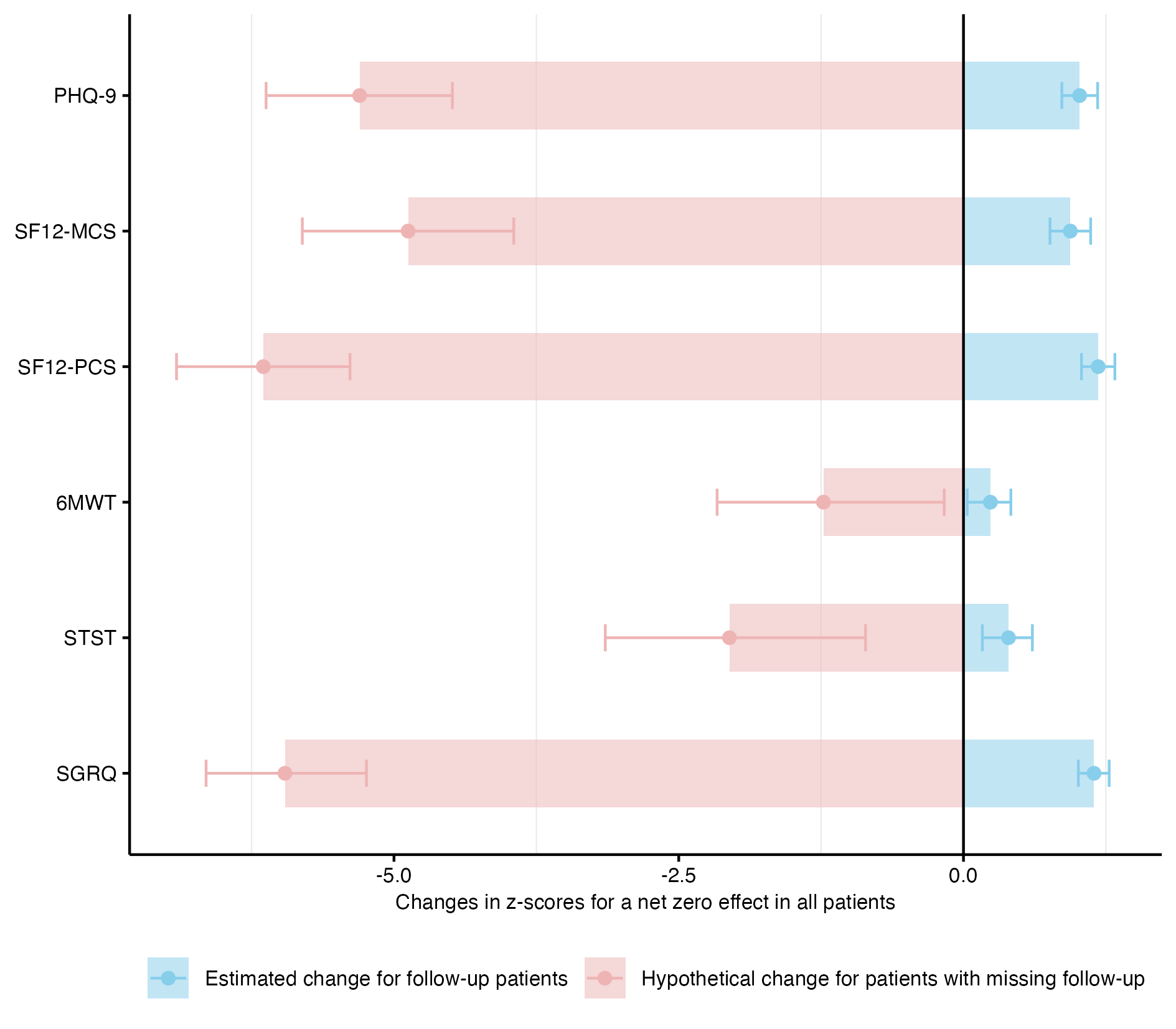
**

As a simulation exercise, we estimate for each outcome the change in QoL that would be needed in patients who died or did not return for a follow-up visit at the end of treatment to offset the positive QoL changes estimated for patients with follow-up visits, i.e. a net zero effect or an average change in the z-scores of zero. We compute this as $b\cdot n/m$, where *b* is the model-estimated effect, *n* is the number of patients with and *m* is the number of patients without follow-up visits by the end of treatment.

**Figure S10: Estimated change in QoL z-score (median as dot, 95%-CrI as lines) per quantile of the QoL score distribution at the end of tuberculosis treatment by patient characteristic.** PHQ-9 and SGRQ scores were transformed so that for all outcomes, a positive estimate indicates a positive association of the patient characteristic with QoL.

**
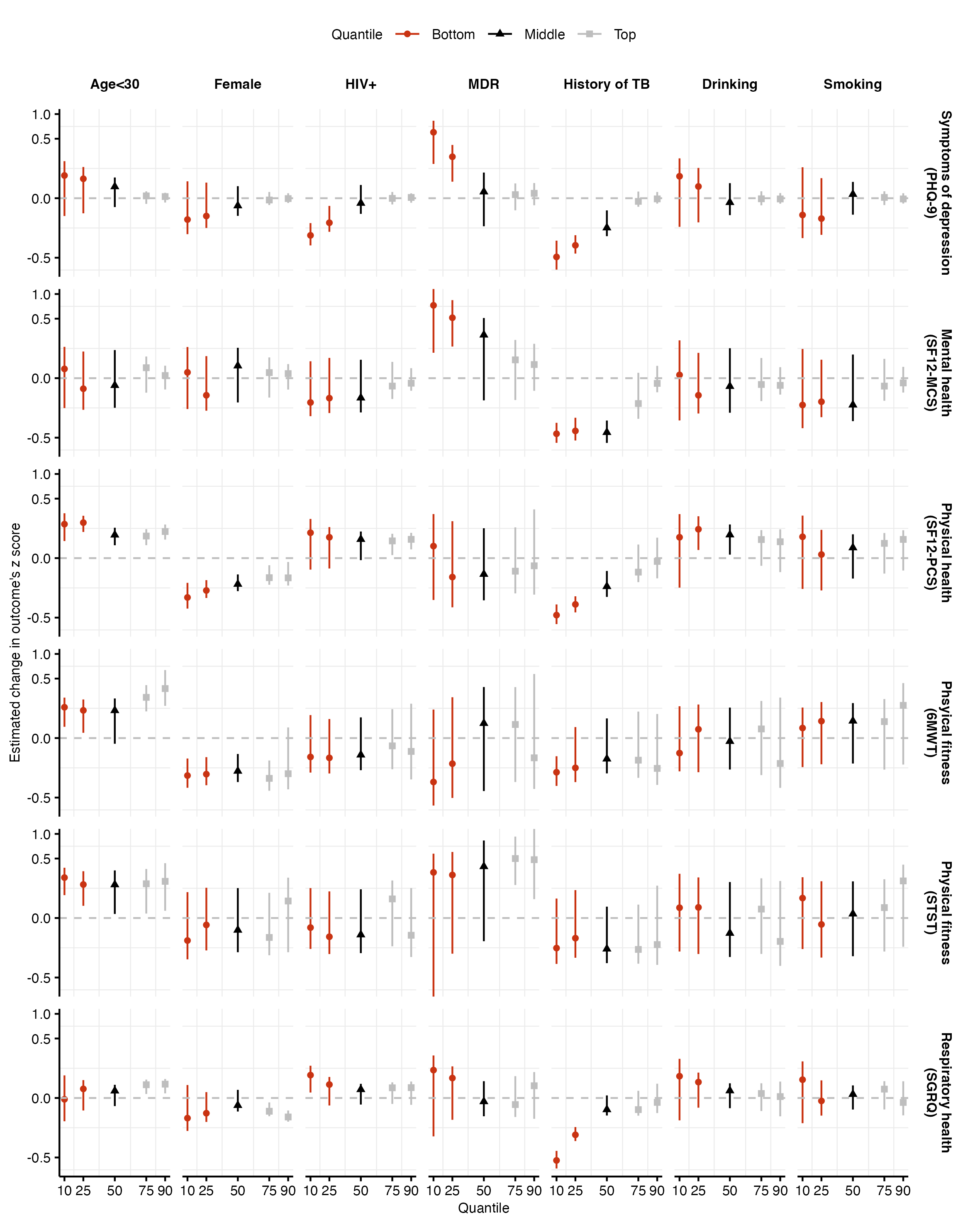
**

MDR, multi-drug resistant tuberculosis; TB, tuberculosis

To investigate what patients did not improve during tuberculosis treatment, we estimated the association of patient characteristics with different quantiles of the QoL score distribution at the end of tuberculosis treatment using quantile regression. We adjusted the model for the baseline QoL scores, non-fatal serious adverse events, and mental health treatment. Again, we excluded missing scores of patients who died or did not return for a follow-up visit.
